# Intranasal trivalent candidate vaccine induces strong mucosal and systemic immune responses against *Neisseria gonorrhoeae*

**DOI:** 10.3389/fimmu.2024.1473193

**Published:** 2024-11-26

**Authors:** Qin Lu, Hui Yang, Yanfeng Peng, Zeling Dong, Pujing Nie, Guangli Wang, Shilu Luo, Xun Min, Jian Huang, Meirong Huang

**Affiliations:** ^1^ School of Laboratory Medicine, Zunyi Medical University, Zunyi, Guizhou, China; ^2^ Department of Laboratory Medicine, Affiliated Hospital of Zunyi Medical University, Zunyi, Guizhou, China; ^3^ Department of Blood Transfusion, Affiliated Hospital of Zunyi Medical University, Zunyi, Guizhou, China

**Keywords:** *Neisseria gonorrhoeae*, MetQ, neisserial heparin binding antigen, adhesion and penetration protein, nasal immunization

## Abstract

The spread of multidrug-resistant strains of *Neisseria gonorrhoeae* poses a great challenge in gonorrhea treatment. At present, vaccination is the best strategy for gonorrhea control. However, given the extensive antigenic variability of *N. gonorrhoeae*, the effectiveness of monovalent vaccines is limited. Therefore, increasing the coverage of vaccination by using a multivalent vaccine may be more effective. In this study, a trivalent vaccine comprising three conserved antigens, namely, the App passenger domain, MetQ, and neisserial heparin binding antigen (NHBA), was constructed, and its protective effect was evaluated. Trivalent vaccines induced stronger circulating IgG and IgA antibody responses in mice than monovalent vaccines, in addition to eliciting Th1, Th2, and Th17 immune responses. Antiserum generated by the trivalent vaccine killed *N. gonorrhoeae* strains (homologous FA1090 and heterologous FA19), exhibiting superior bactericidal capacity than NHBA and MetQ vaccine antisera against *N. gonorrhoeae*, but similar capacities to those of the App vaccine antiserum. In addition, the trivalent vaccine antiserum achieved greater inhibition of *N. gonorrhoeae* FA1090 strain adherence to ME-180 cells compared to that elicited by the monovalent vaccine antiserum. In a mouse vaginal infection model, the trivalent vaccine was modestly effective (9.2% decrease in mean area under curve compared to the pCold-TF control mice), which was somewhat better than the protection seen with the monovalent vaccines. Our findings suggest that recombinant multivalent vaccines targeting *N. gonorrhoeae* exhibit advantages in protective efficacy compared to monovalent vaccines, and future research on multivalent vaccines should focus on optimizing different antigen combinations.

## 1 Introduction

*Neisseria gonorrhoeae* causes the sexually transmitted disease gonorrhea, threatening global health (1). Each year, 87 million people are estimated to be infected with *N. gonorrhoeae* globally (2), with the infection leading mainly to urethritis in men and cervicitis in women. In women, untreated infections may result in pelvic inflammatory diseases and infertility (3). Additionally, gonorrhea increases the risk of contracting and transmitting the human immunodeficiency virus (4). *N. gonorrhoeae* has been reported to be resistant to all antimicrobials recommended for gonorrhea treatment (5). Extensively drug-resistant strains of *N. gonorrhoeae* with high levels of resistance to ceftriaxone and azithromycin have been developed in the United Kingdom and Australia (6, 7). The *N. gonorrhoeae* FC428 strain, which is resistant to first-line therapeutic agents such as ceftriaxone and azithromycin, has spread worldwide (8). Thus, the World Health Organization has identified drug-resistant *N. gonorrhoeae* as an urgent threat to public health (9). As treatment failure due to antibiotic resistance is common, an effective vaccine is required to prevent and treat gonorrhea (1).

Since the 1970s, the protective efficacy of several *N. gonorrhoeae* vaccines have been explored in clinical trials, including a partially inactivated whole-cell vaccine, a gonococcal pilus vaccine, and a gonococcal outer membrane (OM) vaccine enriched in gonococcal porin. However, none of these vaccines have been successful. In recent years, novel strategies, such as reverse vaccinology (10), “omics”, and bioinformatics have been employed to discover *N. gonorrhoeae* vaccine antigens. As a result, *N. gonorrhoeae* candidate vaccine antigens have been evaluated in preclinical settings, including the gonococcal lipooligosaccharide epitope 2C7 (11) and some surface membrane proteins (1). For example, MetQ, the methionine-binding component of an ATP-binding cassette transporter system, is a highly conserved lipoprotein in different strains of *N. gonorrhoeae* (12–14). In addition to its function in methionine transportation, MetQ also affects *N. gonorrhoeae* adhesion to epithelial cells, invasion, and survival in primary monocytes, macrophages, and human serum (15). The rMetQ-CpG vaccine significantly accelerates the clearance rate of gonococcal infections in immunized mice and reduces the bacterial load (16). Recent studies have reported that serum group B *N. menigitidis* OM vesicle (OMV) vaccines, including MeNZB, 4CMenB, MenVA-MENGOC-BC, and MenBvac, elicit moderate cross-protection against *N. gonorrhoeae* (1, 17, 18), which has reignited interest in *N. gonorrhoeae* multivalent OMV vaccines. A gonococcal vaccine composed of OMVs and microencapsulating IL-12 elicits Th1-driven immunity, generates both circulating and local antibodies in the genital tract, and confers resistance to vaginal gonococcal infections upon intravaginal immunization (19). Additionally, the nasal immunization route likewise elicits immune responses in the genital tract and confers resistance to vaginal infection with multiple *N. gonorrhoeae* strains (20). However, OMVs are derived from the bacterial OM and contain a variety of protein components, which are highly variable during production and expensive to extract (21). Increasing vaccine coverage through co-vaccination with multiple key surface antigens to minimize the likelihood of immune escape and the selection of resistant mutants may be effective for developing an efficient gonorrhea vaccine (22). Gulati et al. showed that a chimeric molecule comprising NGO0265 and FtsN adjuvanted with GLA-SE elicits an IgG response with broad anti-gonococcal bactericidal activity and attenuates gonococcal colonization in a complement-dependent manner (23). In addition, Zhu et al. found that the killing ability of anti-NGO0690 and anti-NGO1701 mixed serum against *N. gonorrhoeae* F62 strain was significantly improved compared with a single serum, with an additive effect (24). They further constructed a trivalent vaccine consisting of recombinant proteins NGO0690, NGO0948, and NGO1701 that strongly induced serum bactericidal antibodies against several *N. gonorrhoeae* strains (22). Consequently, multivalent vaccines may be a promising research direction for gonorrhea prevention.

The adhesion and penetration protein (App), an autotransporter with serine protease activity in *N. gonorrhoeae*, is a conserved virulence factor localized on the bacterial surface and involved in bacterial adhesion, invasion and colonization (14, 25). Our previous study suggested that, within the intranasal mucosa, the passenger domain of the App protein is immunoprotected against gonococcal infections (26). Neisserial heparin binding antigen (NHBA) is a surface-exposed lipoprotein that binds to heparin and heparan sulfate proteoglycans on the surface of host epithelial cells (27). Recombinant NHBA is highly immunogenic, and antibodies against NHBA mediate the killing of gonococci through serum bactericidal and opsonophagocytic activities. These antibodies also prevent the functional activity of NHBA by reducing heparin binding and adherence to cervical and urethral epithelial cells (28). Since research on gonococcal multivalent recombinant vaccines remains in its infancy, it is essential to explore and optimize more antigen combinations. Based on the important roles of MetQ, App, and NHBA in the adhesion and invasion of *N. gonorrhoeae*, this study aimed to explore the protective effects of these three protein combinations against gonorrhea.

*N. gonorrhoeae* usually colonizes the genitourinary system (3). However, most current gonorrhoeae vaccines are systemic and do not elicit mucosal immunity to effectively prevent or reduce transmission (29, 30). Therefore, in the present study, we evaluated the *in vivo* immune response and protection against gonococcal infections induced by a trivalent combined-protein vaccine consisting of the App passenger domain, NHBA, and MetQ proteins in combination with the mucosal adjuvant cholera toxin B subunit (CTB), delivered via nasal immunization. Our findings provide a foundation for developing multivalent vaccines and for further characterization of these antigens.

## 2 Materials and methods

### 2.1 Bacterial strains and growth conditions

*N. gonorrhoeae* standard strains FA1090 (ATCC700825) and FA19 (BAA-1838) and two *N. gonorrhoeae* strains from clinical isolates (Department of Medical Laboratory, Affiliated Hospital of Zunyi Medical University) were plated overnight on gonococcal base (GCB) agar (Oxoid, Basingstoke, UK) at 37°C with 5% CO_2_. To obtain sialylation of *N. gonorrhoeae*, a final concentration of 50 μg/mL CMP-N-acetylneuraminic acid (CMP-NANA) (Sigma) was added to gonococcal base liquid (GCBL) medium and grown at 37°C for 18 h in an atmosphere of 5% (v/v) CO_2_ (30, 31). In some experiments, bacteria grown for 18–24 h were resuspended in phosphate-buffered saline (PBS) using a Sensititre nephelometer to prepare the corresponding bacterial suspensions.

### 2.2 Vaccine design and construction

Sequences of the App passenger domain (*ngo*2105), NHBA protein (*ngo*1958), and MetQ protein (*ngo*2139) were obtained from the NCBI database (GenBank: NC_002946.2). Genes encoding the passenger domain (43–1190 aa), NHBA protein (1–429 aa), and MetQ protein (23–288 aa) fragments were amplified from *N. gonorrhoeae* FA1090 through polymerase chain reaction using the primers listed in **Supplementary Table S1**. The above gene fragments were amplified and cloned into the pCold-TF vector (plasmid map is shown in **Supplementary Figure S1**) to obtain template plasmids pCold-TF-*passenger*, pCold-TF-*nhba*, and pCold-TF-*metQ*.

### 2.3 Expression and purification of target proteins

The pCold-TF-*passenger*, pCold-TF-*nhba*, and pCold-TF-*metQ*, and pCold-TF plasmids were transformed into *E. coli* BL21 (DE3) cells using heat shock. Bacterial strains transformed with recombinant plasmids were cultured on Luria–Bertani agar plates supplemented with ampicillin (100 µg/mL) and then incubated at 37°C for 12–15 h. A single colony was inoculated into Luria–Bertani broth supplemented with ampicillin in a conical flask and then incubated at 37°C for 12–15 h with shaking at 250 *rpm*. When the bacterial culture reached an optical density of 0.60 at 600 nm (OD_600_), 0.05 mM isopropyl beta-D-thiogalactoside was added to induce recombinant protein expression, which continued for 10 h at 15°C. Recombinant proteins were purified using a Ni^2+^-nitrilotriacetic acid column, and imidazole was removed. Lipopolysaccharide was removed from the recombinant protein preparations using an EtEraser™ Endotoxin Removal Kit (Xiamen Bioendo Technology Co. Ltd, Xiamen, China). After the recombinant protein was treated with endotoxin removal, LPS content in the recombinant protein was detected using a gram-negative lipopolysaccharide detection kit (Xinuo Biopharmaceutical Co, LTD, Tianjin, China), which was lower than 0.5 EU/mL. Protein purity was analyzed via 10% sodium dodecyl sulfate-polyacrylamide gel electrophoresis (SDS-PAGE) and Coomassie Blue Fast staining (Epizyme, China). The protein concentration was determined using a BCA kit (Solarbio, Beijing, China), followed by storage at −80°C until use.

### 2.4 Immunization of mice

Female BALB/c mice (6–8 weeks old) of specific-pathogen-free (SPF) grade were purchased from the Experimental Animal Center of Zunyi Medical University (Zunyi, China). The mice (n = 6) were randomly divided into six groups: App + CTB, NHBA + CTB, MetQ + CTB, App + NHBA + MetQ (trivalent + CTB), pCold-TF + CTB, and PBS + CTB. Mice were immunized intranasally on days 0, 14, 28, and 42 (32). Then, 10 µg of App, NHBA, MetQ, or pCold-TF protein or 10 µg each of App, NHBA, MetQ, or their mixture (App + NHBA + MetQ) were suspended in 20 μL of PBS and mixed with 10 μg of CTB adjuvant (Absin, Shanghai, China) to a total volume of 30 μL for mouse immunization. Tail vein blood was collected from the mice 1 week after each vaccination, and vaginal secretions were collected on days 35 and 49. For vaginal secretions, 50 μL of sterile PBS was aspirated into the posterior vaginal fornix three to five times, repeated twice, and the fluids were combined. Finally, after centrifugation, the supernatants of the serum and vaginal secretions were stored at −80°C until further analysis.

### 2.5 Native target protein expression in different *N. gonorrhoeae* strains

*N. gonorrhoeae* FA1090, FA19, and the two clinical strains were inoculated on GCB plates and incubated at 37°C for 24 h. Colonies were picked and placed in PBS and adjusted to 0.5 McFarland units. The bacterial precipitates were washed with PBS and added to 1× loading buffer, then boiling at 100°C for 15 min. Bacterial lysates were separated via SDS-PAGE and transferred to polyvinylidene fluoride membranes. The expression of App passenger, NHBA, and MetQ in different *N. gonorrhoeae* strains were analyzed through immunoblotting using App, NHBA, or MetQ antisera as the primary antibodies (1:2,000) and horseradish peroxidase-labelled goat anti-mouse IgG (ZSGB-Bio, China) as the secondary antibody (1:5,000). Finally, a high-sensitivity chemiluminescent substrate detection kit (Epizyme, Cambridge, MA, USA) was used to analyze App, NHBA, and MetQ expression in different *N. gonorrhoeae* strains.

### 2.6 Mouse antibody titers and antibody typing

Antibody titers in serum and vaginal secretions were assessed using enzyme-linked immunosorbent assays (ELISAs). Nunc-Immuno plates were coated with purified antigen (96 wells of purified antigen for App, NHBA, MetQ, or pCold-TF at 10 μg/mL), incubated overnight at 4°C, and washed with PBS-T (PBS containing 0.1% Tween 20). Antisera from the immunized groups were diluted with blocking buffer (100 μL/well) and incubated at 37°C for 1 h in the wells. The plates were then washed three times with PBS-T. Horseradish-peroxidase-labelled goat anti-mouse IgG, IgG1, IgG2a, IgG2b, IgG3, and IgA were diluted at 1:5,000 and added to the plates, which were left to stand for 1 h at 37°C. The plates were washed three times, and a 3,3’,5,5’-tetramethylbenzidine solution (Solarbio) was added for 30 min for color development. The reaction was terminated by adding a termination solution (Solarbio). Absorbance was recorded at 450 nm, and the antibody titer was defined as the highest dilution of the sample with an absorbance value 2.1 times that of the negative control.

### 2.7 Serum bactericidal activity

For the serum bactericidal assay (SBA) (33), *N. gonorrhoeae* FA1090 and FA19 were inoculated into GCB plates and incubated for 18–24 h at 37°C with 5% CO_2_. To obtain sialylation of *N. gonorrhoeae* strain FA1090 was cultured in GCBL medium (containing 50 μg/mL CMP-NANA) in 5% (v/v) CO_2_ at 37°C for 18 h. The number of selected colonies was adjusted to 4 × 10^4^ colony-forming units (CFU)/45 μL. Then, 45 μL of heat-inactivated antisera at different dilution titers (mixture of three mouse sera samples) was added, and the plates were incubated at 37°C with 5% CO_2_ for 15 min. Thereafter, 25% human serum was added as a complement source, followed by incubation at 37°C, 5% CO_2_ for 30 min. The entire reaction mixture was diluted and spread onto GCB plates, with the CFU counted on the following day. The SBA bactericidal titers had the highest antibody dilutions, which resulted in more than 50% *N. gonorrhoeae* killing. The untreated group (0, without serum and complement), the lowest dilution of PBS-immunized group antiserum (PBS, with complement) and pCold-TF-immunized group antiserum (pCold-TF, with complement) were set as controls. The bacterial survival rate of the untreated group (0, without serum and complement) was set to 100%, and the bacterial survival rate of each group was calculated by the number of colonies in each serum group/number of colonies in the untreated group ×100%.

### 2.8 Antibody adherence inhibition assay

As described previously (26), an antibody-mediated adhesion inhibition assay was performed using human cervical cancer epithelial cells (ME-180, ATCC HTB33, Fenghui Biotechnology Co, Ltd, Hunan, China). ME-180 cells were cultured in RPMI 1640 with 10% fetal bovine serum for 24 h to form a monolayer of fused cells in 24-well tissue culture plates. Bacterial suspensions were prepared by inoculating *N. gonorrhoeae* FA1090 onto GCB plates and incubating overnight. Colonies with pili were then selected. The bacteria were pre-incubated with heat-inactivated antiserum in RPMI 1640 medium (HyClone, Logan, UT, USA) for 30 min at 37°C. ME-180 cells were washed three times with PBS, and the antiserum-pre-treated bacterial suspension was added at a multiplicity of infection of 10:1 to the ME-180 cells. This was followed by co-incubation at 37°C and 5% CO_2_ for 3 h. To remove the non-adherent bacteria, we washed the solution three times with PBS. The ME-180 cells were lysed with 1% saponin. The lysates were serially diluted and plated on GCB plates to determine the bacterial number of CFU. Adhesion rates were calculated as the ratio of the number of CFU that adhered to the cells to the initial number of CFU.

### 2.9 Whole-cell ELISA

Whole-cell ELISA experiments were carried out as described (15). The ELISA was conducted using 96-well MaxiSorp plates (Nunc) coated with *N. gonorrhoeae* FA1090 strains (1×10^7^ CFU). After coating, the plates were washed with PBS and blocked with PBS containing 1% bovine serum albumin (BSA) for 1 hour. Following blocking, the plates were incubated with antibody (diluted 1:1,000) for 1 hour at 37°C, 5% CO_2_. After washing three times with PBS, the plates were incubated with a secondary antibody (HRP-conjugated anti-mouse, diluted 1:2,000) for 1 hour. After additional washes, the plates were developed using tetramethylbenzidine (TMB) solution (Solarbio). The reaction was terminated by adding 1 volume of 1 M HCl. Absorbance was measured at 450 nm using a Victor3 plate reader. Relative binding capacity was calculated as A450_sialylation_/A450_non-sialylation_ for each antiserum.

### 2.10 Assay of cytokine levels

One week after the last immunization, three mice were randomly selected from each group. Mouse spleens were crushed and resuspended through filtration in RPMI 1640 to prepare splenocyte suspensions that were supplemented with 10% fetal bovine serum, 10 kU/mL penicillin, 10 mg/mL streptomycin, and 25 μg/mL amphotericin B. The suspension was incubated at room temperature for 24 h. The cells were added to 24-well cell culture plates (1 mL/well) at 5 × 10^6^ cells/mL. Finally, splenocytes from immunocompetent mice were stimulated by adding 10 μg of purified App, NHBA, MetQ, App + NHBA + MetQ (Trivalent), or pCold-TF proteins to PBS and cultured for 72 h at 37°C and 5% CO_2_. The culture medium supernatant was collected, and the levels of IL-17A and IFN-γ were determined using cytokine assay kits (Proteintech, Rosemont, IL, USA).

### 2.11 Mouse immunity and challenge studies

SPF-grade female BALB/c mice aged 6 to 8 weeks (Experimental Animal Centre, Zunyi Medical University) were randomly divided into the following six groups (n = 6): App, NHBA, MetQ, App + NHBA + MetQ (Trivalent), pCold-TF, and PBS. Mice were vaccinated via intranasal inoculation on days 0, 14, 28, and 42. The vaccine contained 10 μg of antigen and 10 μg of CTB (CTB adjuvant:antigen = 1:1; total volume 30 μL). After the final immunization, pro-estrus mice were administered subcutaneous injections of 0.5 mg of sesame-oil-soluble estradiol on days −2, 0 (day of bacterial challenge), and +2 to increase their susceptibility. Mice were treated with antibiotics to prevent the overgrowth of commensal flora. FA1090 (1 × 10^8^ CFU/mL) was prepared, and mice were infected with 20 μL of this solution to achieve an infective dose of approximately 2 × 10^6^ CFU/mouse. Each group of mice was vaginally inoculated with vaginal secretions (vaginal rinse with 50 μL of normal saline, repeated twice), which were collected daily to dilute the smear for colony counting and observe the clearance of *N. gonorrhoeae* colonization.

### 2.12 Statistical analysis

Comparisons between two independent groups were performed using the Student’s t-test. Antibody titers were analyzed using Šídák’s multiple comparison test. Kaplan–Meier plots were used to analyze the time needed for infection clearance. The log-rank (Mantel–Cox) test was used to compare the Kaplan–Meier curves of the groups. The AUC comparisons should be made by One-way ANOVA and Dunn’s multiple comparisons test.

## 3 Results

### 3.1 Preparation and characterization of the trivalent vaccine

**Figure 1:**
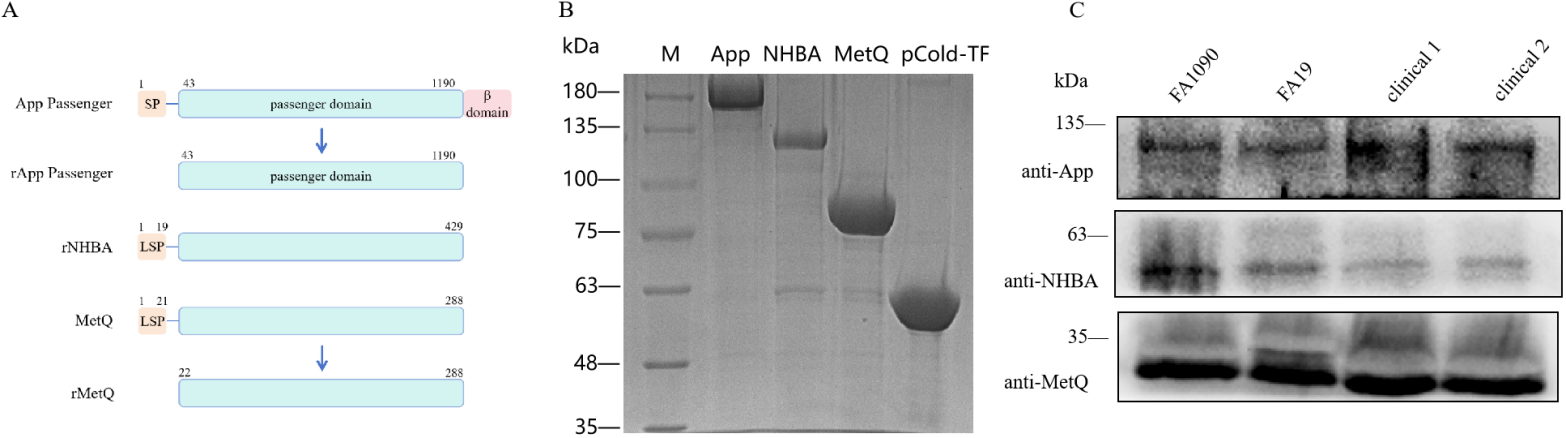
Expression and identification of trivalent vaccine component proteins. **(A)** Schematic of the protein domains. To generate rApp passenger antigen, which lacks the signal peptide (SP) and β domain, we engineered a partial passenger domain (43–1190 aa). To generate rNHBA antigen, the lipoprotein signal peptide (LSP)-containing *nhba* gene was engineered to produce a full-length NHBA recombinant protein. To generate rMetQ antigen, the *metQ* gene, encoding a full-length MetQ protein, was engineered to produce a recombinant protein that lacks the LSP. **(B)** Recombinant proteins were separated using 7.5% sodium dodecyl sulfate-polyacrylamide gel electrophoresis. Lane M: Marker, lane 1: App passenger domain, lane 2: full-length NHBA protein, lane 3: truncated MetQ protein, lane 4: pCold-TF-tagged protein. These recombinant proteins were expressed as a fusion that comprised Trigger factor plus three peptides that contained cleavage sites for HRV3C protease, Thrombin, and Factor Xa. **(C)** Western blotting analysis of App, NHBA, and MetQ protein expression in different gonococcal strains using antibodies generated against the recombinant proteins.

To construct the trivalent vaccine, we used the App protein passenger domain, full-length NHBA, and truncated MetQ with the signal peptide region removed as vaccine components (**Figure 1A**). These components were expressed in *E. coli* BL21 with the tag protein “trigger factor” in the pCold-TF vector, resulting in pure bands of the target proteins at relative molecular masses of 180, 130, 90, and 55 kDa, respectively (**Figure 1B**). Polyclonal antibodies were prepared by immunizing mice with the three tagged recombinant proteins. Western blotting showed that the polyclonal antibodies specifically recognized natively expressed App, NHBA, and MetQ in two laboratory strains, FA1090 and FA19, as well as in randomly selected clinical isolates (**Figure 1C**). The genetic sequences of 100 strains of *N. gonorrhoeae* showed 99.93% 96.37%, and 99.88% conservation of *app*, *nhba* and *metQ* genes, respectively (**Supplementary Tables S2–S4**), suggesting that App, NHBA, and MetQ are conserved among different strains.

### 3.2 Circulating and genital antibody responses

**Figure 2:**
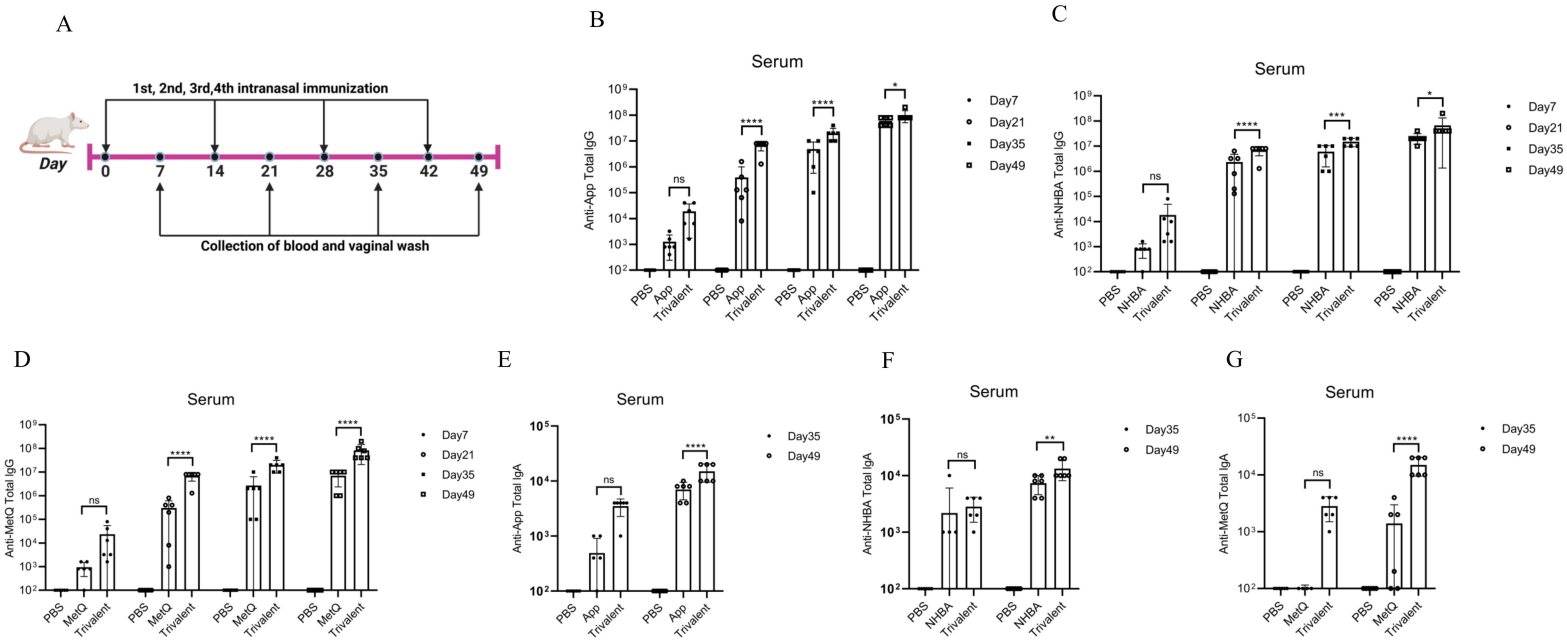
Comparison of serum antibody responses to nasal immunization in mice. **(A)** Timeline of mouse immunity. **(B–D)** On days 7, 21, 35, and 49 post-immunization, the serum titers of anti-App, anti-NHBA, and anti-MetQ IgG antibodies in each group were determined using enzyme-linked immunosorbent assay (ELISA). **(E–G)** On days 35 and 49 of immunization, anti-App, anti-NHBA, and anti-MetQ IgA serum antibody titers were determined using ELISAs. Data are presented as the mean ± standard error of the mean (SEM), n = 6, compared to the monovalent group (Šídák’s multiple comparisons test), ns: non-significant, *P* > 0.05; *P ≤ 0.05; **P ≤ 0.01; ***P ≤ 0.001; ****P ≤ 0.0001.

**Figure 3:**
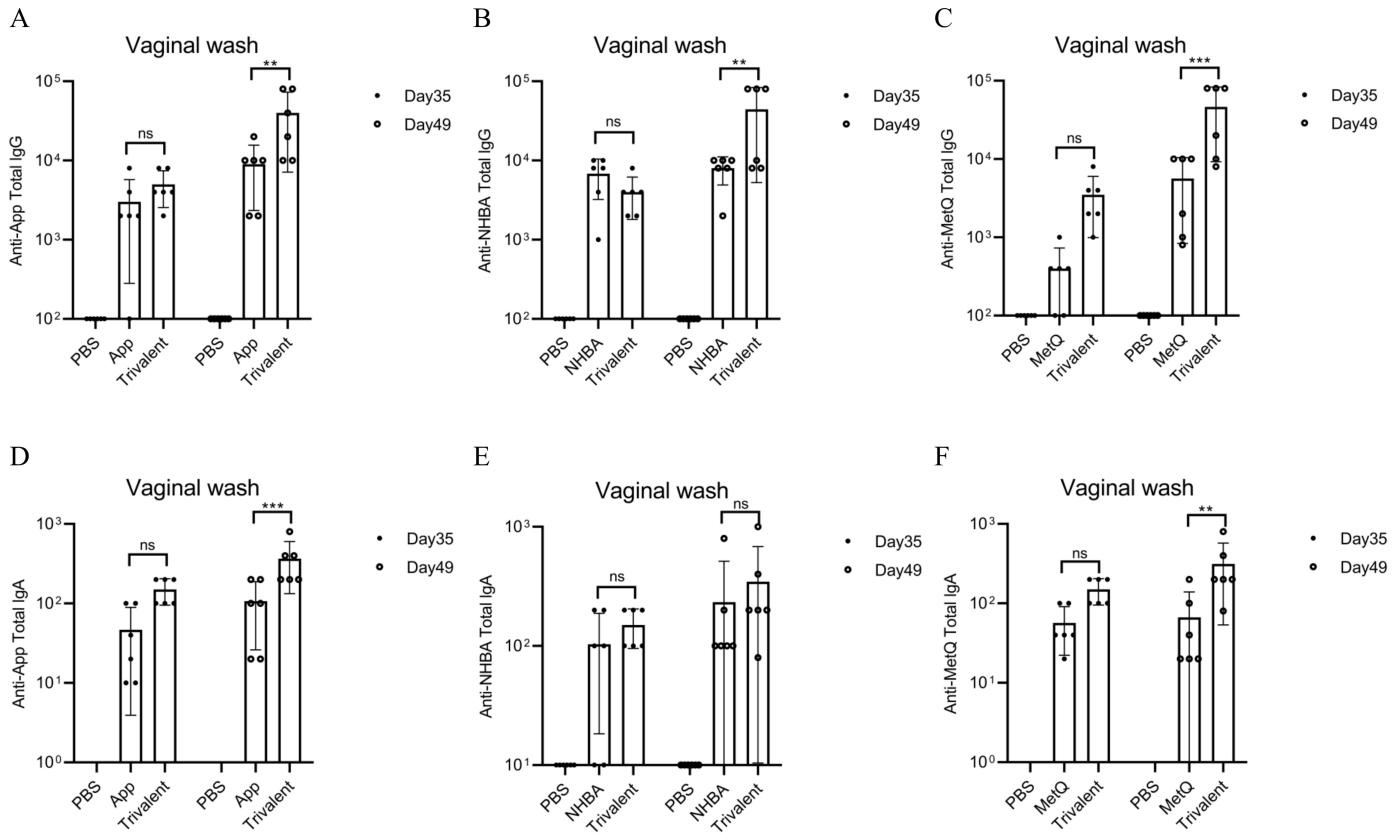
Comparison of vaginal antibody responses after nasal immunization in mice. On days 35 and 49 post-immunization, anti-App, anti-NHBA, anti-MetQ IgG **(A–C)**, and IgA **(D–F)** antibody titers in vaginal wash fluid from each group were determined using ELISAs. Data are presented as the mean ± SEM, n = 6, compared to the monovalent group (Šídák’s multiple comparisons test), ns, non-significant, *P* > 0.05; **P ≤ 0.01; ***P ≤ 0.001.

Mice were immunized with tagged recombinant proteins intranasally on days 0, 14, 28, and 35, with serum and vaginal secretions collected to determine antibody titers 1 week after each immunization (**Figure 2A**). Overall, mice immunized with the trivalent vaccine tended to exhibit stronger circulating IgG and IgA antibody responses than those immunized with the monovalent vaccine (**Figures 2B–G**). Both IgG and IgA antibodies were significantly induced in vaginal washes (**Figures 3A–F**). Except for anti-NHBA IgG, the trivalent vaccine group had significantly higher IgG and IgA antibody titers in the vaginal washes after the fourth immunization than those in the monovalent vaccine group.

### 3.3 Th1, Th2, and Th17 immune responses

**Figure 4:**
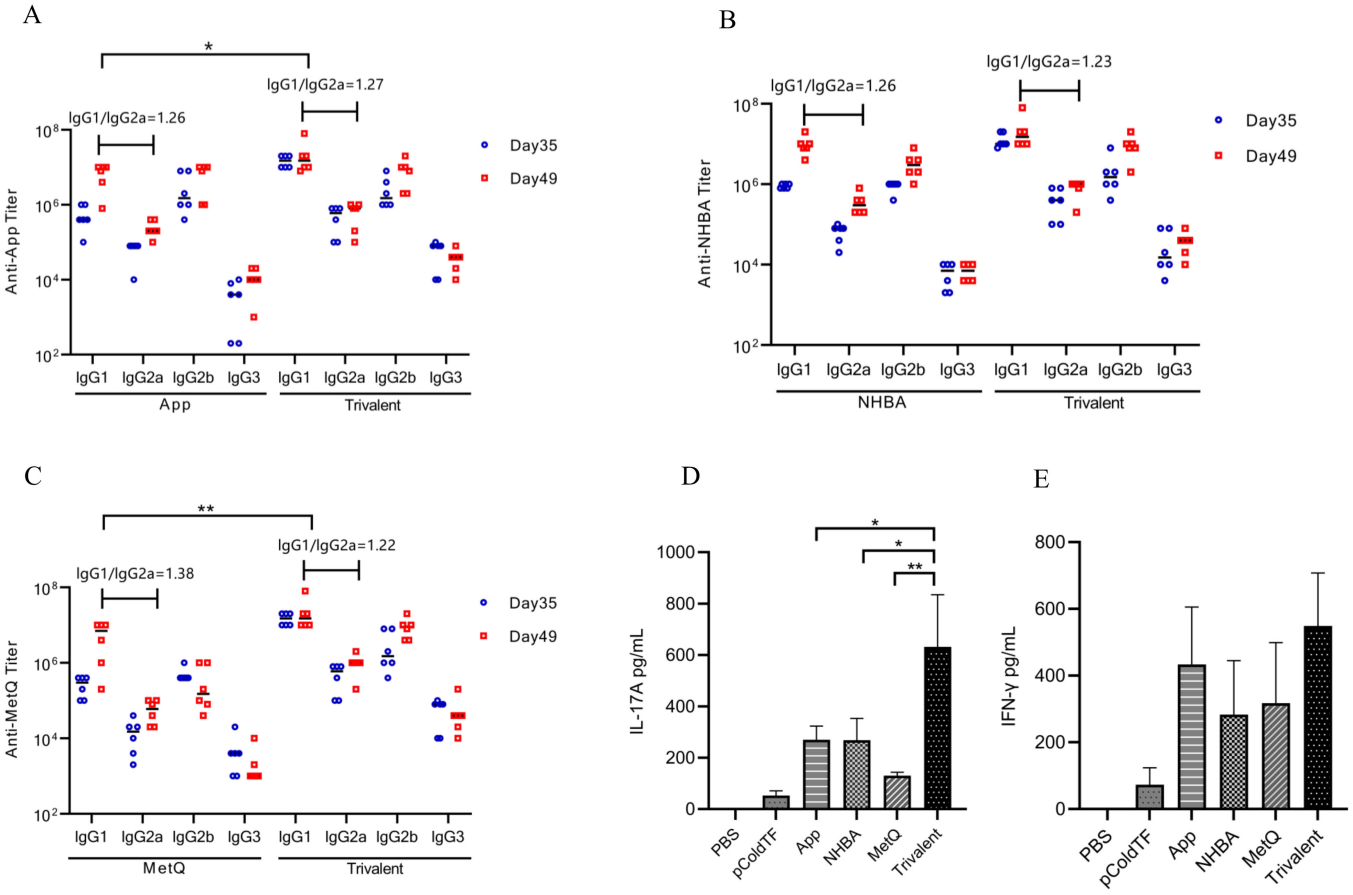
Serum antibody subtype and spleen cytokine analysis. **(A–C)** Antigen-specific IgG1, IgG2a, IgG2b, and IgG3 titers in serum were analyzed on days 39 and 49 post-immunization, and the IgG1/IgG2a ratio was calculated using antibody titer data from day 49 post-immunization (n = 6). **(D, E)** On day 49 post-immunization, three mice in each group were randomly selected for the isolation of splenocytes, which were stimulated with 10 μg of the corresponding recombinant proteins for 72 h, whereafter the supernatant was analyzed for IL-17A and IFN-γ levels. Data are presented as the mean ± SEM, compared to the monovalent group (unpaired Student’s t-test), *P ≤ 0.05, **P ≤ 0.01.

The IgG isotype indirectly reflects changes in the Th1/Th2 balance (26). In mice, Th1-type cytokines induce lgG2a, whereas Th2-type cytokines induce lgG1 antibody production. After the final immunization, higher titers of each IgG isotype were observed in the trivalent vaccine group than in the monovalent vaccine group (**Figures 4A–C**). All groups generated high titers of IgG1 antibody responses (IgG1 > IgG2b > IgG2a > IgG3) and had IgG1/IgG2a ratios > 1. These results suggest that mice in all groups exhibited a bias toward Th2-type humoral immune responses. The IFN-γ and IL-17A levels were significantly higher in spleen leukocyte supernatants from trivalent vaccine-, App-, NHBA-, and MetQ-immunized mice compared to those in control mice (**Figures 4D, E**). IL-17A levels were significantly higher in the trivalent vaccine group than in the monovalent vaccine group. These results suggested that the trivalent vaccine elicited strong Th1, Th2, and Th17 immune responses.

### 3.4 Bactericidal activity against diverse gonococcal strains

**Figure 5:**
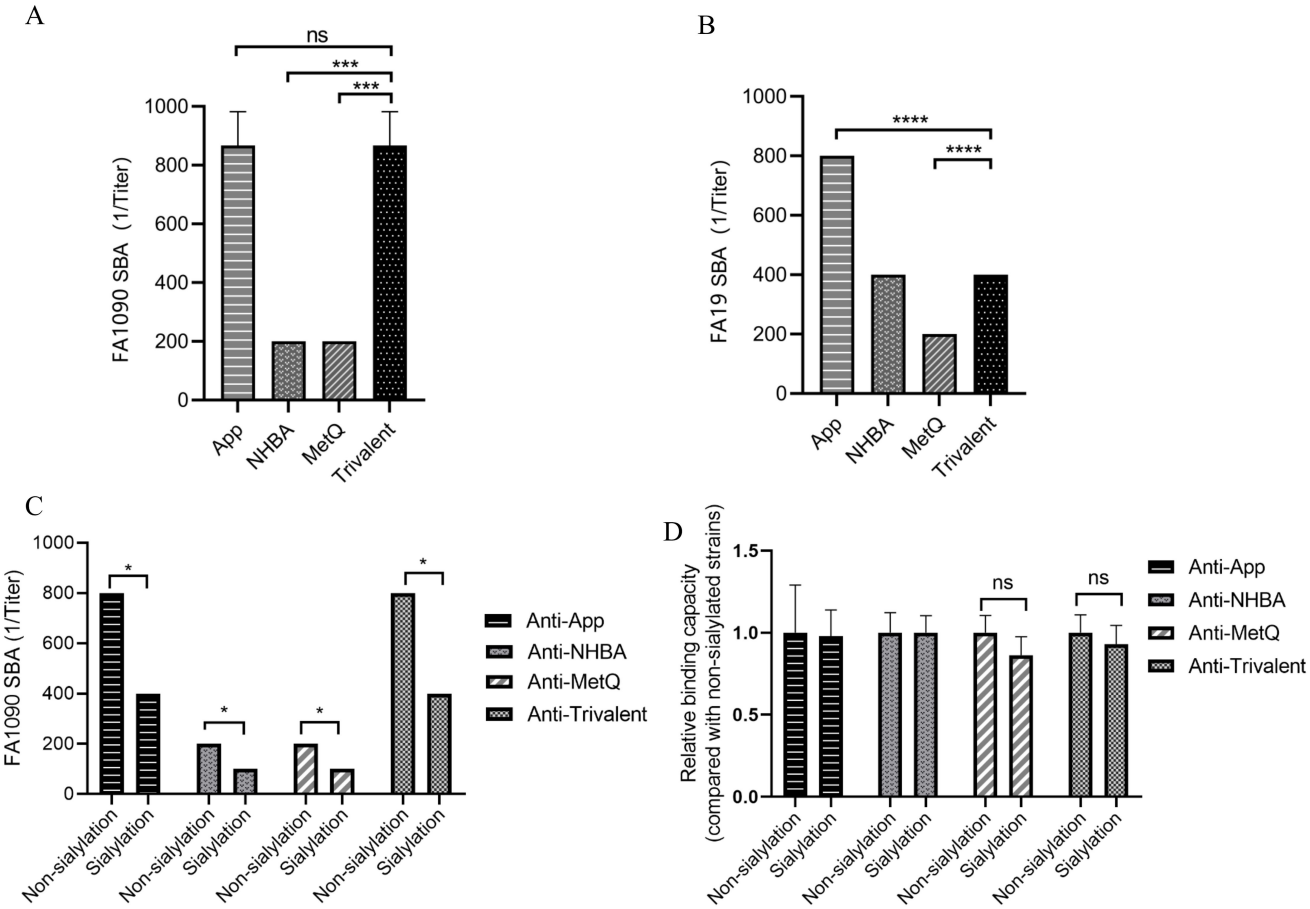
Analysis of antisera for bactericidal activity against *N. gonorrhoeae* strains FA1090 andFA19. For the serum bactericidal experiment, heat-inactivated sera of different dilutions from each immune group were co-incubated with strains FA1090 **(A)** and FA19 **(B)**. **(C)** Bactericidal effect of antiserum on sialylated and non-sialylated *N. gonorrhoeae* FA1090 strains. **(D)** Whole-cell ELISA of sialylated and non-sialylated *N. gonorrhoeae* FA1090 strains. Data are shown as mean ± standard deviation (n ≥ 3), some with identical results, so there were no Error bars. ns, non-significant, *P* > 0.05; *P ≤ 0.05; ***P ≤ 0.001; ****P ≤ 0.0001.

To further investigate the putative protective immune responses, we characterized the functions of antibodies elicited by the trivalent vaccines. We evaluated the bactericidal efficacy of antisera from various immunization groups against three strains, including homologous *N. gonorrhoeae* FA1090 and heterologous FA19. SBA showed that the antiserum from the trivalent vaccine group had stronger bactericidal activity against the three strains than that of the NHBA and MetQ groups, with bactericidal activity range of 100–800 dilution titers (**Figures 5A, B** and **Supplementary Figure S2**). To examine whether sialylation has an effect on bactericidal activity, we cultured strain FA1090 in the presence of CMP-NANA and tested the bactericidal activity of antiserum against both sialylated and non-sialylated gonococci. The results showed that the bactericidal efficiency of several antisera against sialylated gonococci was lower than that of non-sialylated gonococci (**Figure 5C**). We investigated whether sialylation affected the binding of antiserum to *N. gonorrhoeae* FA1090 by whole bacteria ELISA. The results showed no significant differences in the binding ability of the different antisera to sialylated and non-salialylated gonococci (**Figure 5D**).

### 3.5 Gonococcal adhesion to ME-180 cells

**Figure 6:**
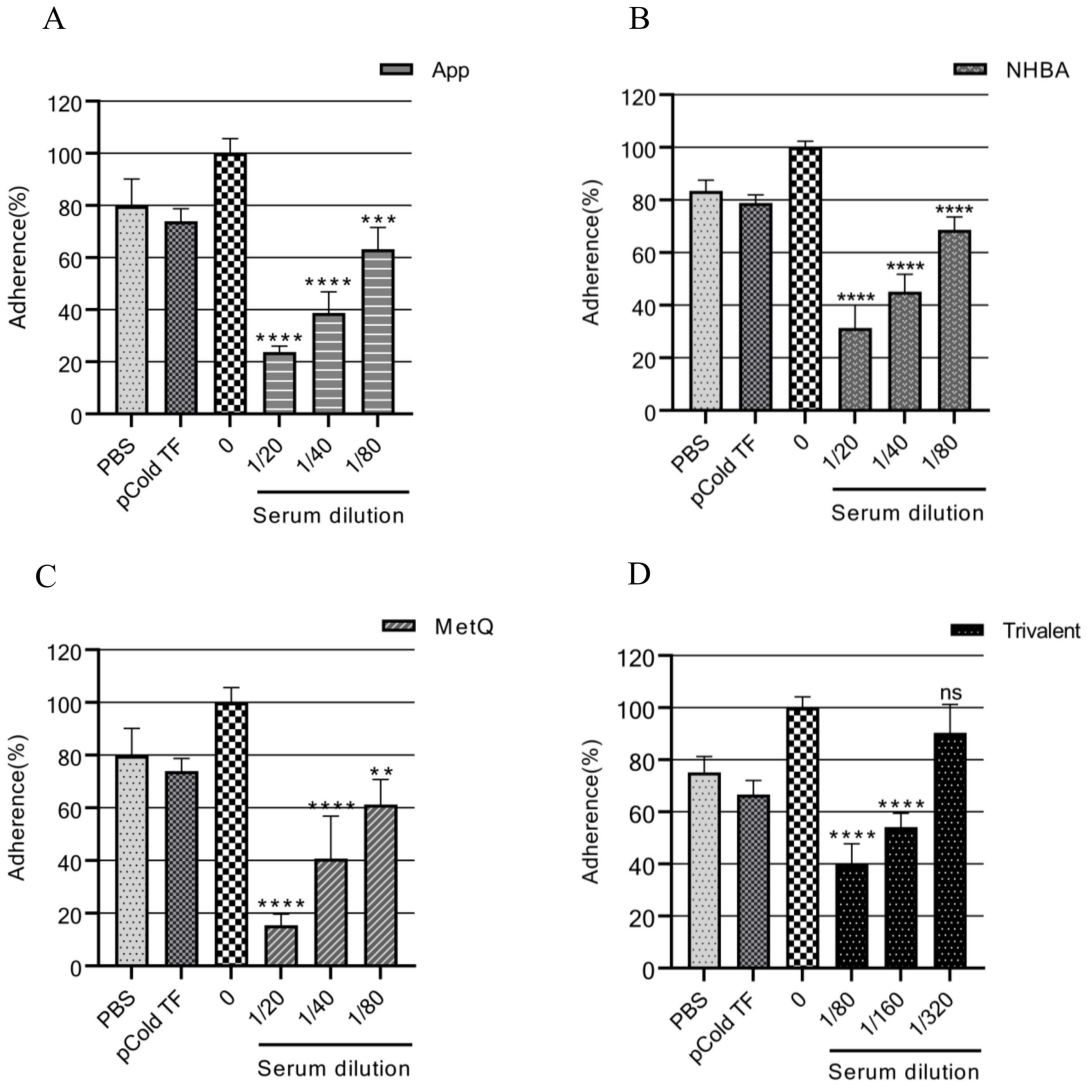
Analysis of the adhesion inhibition effect of antiserum on gonorrhea. FA1090 was pre-incubated with heat-inactivated antiserum of different dilutions in each group for 30 min and then co-incubated with ME-180 cells for 3 h. Antisera from the heat-inactivated PBS and pCold-TF groups were used as controls. The dilution of the PBS and pCold-TF control sera was the lowest dilution of the corresponding experimental group sera. The adhesion rate of *N. gonorrhoeae* in each group was calculated and compared with that in the untreated group (0, 100%). Data are presented as the mean ± SEM (n = 3). ns, non-significant, *P* > 0.05; **P ≤ 0.01; ***P ≤ 0.001; ****P ≤ 0.0001 (unpaired Student’s t-test).

The adhesion of gonococci to epithelial cells is a key mechanism in the establishment of infection (34). Therefore, adhesion experiments were performed on ME-180 cells with strain FA1090 in the presence of antiserum. Our results showed that the antisera from each immunized group inhibited the adhesion of gonococci to ME-180 cells in a concentration-dependent manner (**Figure 6**). When the antisera of the App, NHBA, and MetQ groups were diluted to 1:80, gonococcal adhesion was reduced by 36.76% (*P* < 0.001), 31.37% (*P* < 0.0001), and 38.74% (*P* < 0.01), respectively, whereas the antisera of the trivalent vaccine group reduced gonococcal adhesion by 60.22% at this dilution (*P* < 0.0001). These results showed that the antiserum from the trivalent vaccine group inhibited the gonococcal adhesion rate to a significantly greater extent than antisera from the monovalent vaccine groups.

### 3.6 Clearance of *N. gonorrhoeae* in the vaginal tract

**Figure 7:**
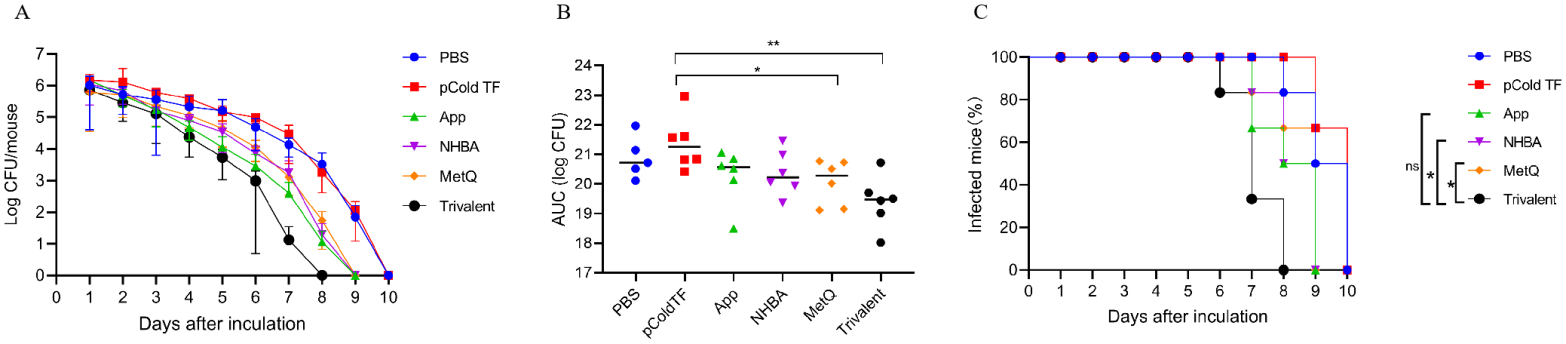
Resistance of each immune group to gonococcal FA1090 infection in the mouse vaginal colonization model. One week after the fourth immunization, pre-estrous mice (n = 6/group) were infected with 2 × 10^6^ colony-forming units (CFU) of the gonococcal FA1090 strain through the vagina, and vaginal secretions were collected every day to count the gonococcal colony number. **(A)** The calculated log10 of CFU per day and plot of the relationship with time. **(B)** Graph displaying the individual area under the curve (AUC) for the daily CFU counts from the colonized mice. **(C)** Graph displaying the removal time curve of gonorrhoeae for each group. Data are presented as the mean ± SEM compared with the monovalent group. Kaplan–Meier curves were analyzed using the Mantel-Cox log-rank test, and AUCs were analyzed using One-way ANOVA and Dunn’s multiple comparisons test, ns, non-significant, *P* > 0.05; *P ≤ 0.05; **P ≤ 0.01.

The efficacy of the trivalent and monovalent vaccines was tested in a mouse model of vaginal gonococcal infection by assessing the daily bacterial loads in vaginal washes and the time to clearance of the infection. There was no significant difference in the number of colonized bacteria between the PBS and pCold-TF controls, whereas the number of colonized bacteria in the monovalent vaccine group was lower than that in the control group (**Figure 7A**). Importantly, the number of colonized bacteria was significantly less in the trivalent vaccine group than in the monovalent vaccine group (**Figure 7A**). The area under the curve (AUC) was significantly lower in the trivalent vaccine group than in the pCold-TF group (**Figure 7B**). Although the AUC of the trivalent vaccine group exhibited a downward tendency when compared with the App, NHBA, and MetQ groups, no statistically significant difference was observed. In terms of the time to infection clearance, mice in the monovalent vaccine group were completely cleared of gonococcal infection on day 9, while for those in the PBS and pCold-TF control groups, clearance was achieved on day 10 (*P* < 0.01, **Figure 7C**). Mice in the trivalent vaccine group were completely cleared of gonococcal infection on day 8, which was significantly faster than clearance in the NHBA and MetQ groups (*P* < 0.01), but not significantly different from that in the App group (*P* = 0.0548, **Figure 7C**). Overall, these results suggest that the trivalent vaccine is somewhat better than the monovalent vaccine in protecting mice against gonococcal infections of the genital tract.

## 4 Discussion

The widespread emergence of antibiotic resistance in *N. gonorrhoeae* poses considerable challenges for the treatment of gonorrhea, with the demand for an effective vaccine becoming increasingly urgent (35). At present, gonorrhea vaccine development is at the level of antigen discovery and the validation of protective immune responses (36, 37). However, given the extensive antigenic variability of *N. gonorrhoeae*, it may be difficult to achieve satisfactory protection using a monovalent vaccine (21, 22). Multivalent vaccines against bacterial and viral pathogens, and even cancer, may provide broader and potentially stronger protection than monovalent vaccines, and targeting multiple epitopes in antigens can also increase strain coverage (38–40). However, multicomponent gonococcal vaccines are relatively understudied (16, 28).

OMV vaccines are multicomponent vaccines (41) that mainly comprise bacterial OM components, which contain key antigens required to induce protective immune responses (42). The OMV-based MeNZB and 4CMenB vaccines can prevent *Neisseria meningitidis* infection (43, 44). *N. meningitidis* and *N. gonorrhoeae* share high genomic homology, with the MeNZB and 4CMenB vaccines being cross-protective against gonorrhea (45, 46). Thus, multivalent vaccines hold promise for protecting against gonorrhea.

In the present study, three antigens were found to be highly conserved among *N. gonorrhoeae* strains. The trivalent vaccine elicited earlier and higher circulating and vaginal IgG and IgA antibody titers than the monovalent vaccine, suggesting stronger immune activation. Serum bactericidal activities reflect immune function (33). Our results showed that antisera from the trivalent vaccine group were able to kill homologous and heterologous *N. gonorrhoeae* strains. However, the bactericidal activities of antisera from trivalent and monovalent vaccine groups differed against different strains (28). This may be caused by the different sensitivities of strains to complement, as well as by possible differences in antigen expression among strains. The bactericidal activities of antisera from the trivalent vaccine group were higher than those of NHBA and MetQ antisera but similar to those of the App group antiserum. Antisera from the trivalent vaccine group did not show additive or synergistic effects on bactericidal activities. A similar phenomenon was observed in the study of Menon et al., when Pfs25 was administered together with Pfs28 or Pfs230C as a mixed-malaria vaccine antigen. The antibody response of each antigen in the mixed vaccine was similar to that of the monovalent vaccine (47). However, the mixed-antigen vaccine did not show a cumulative effect of the two antigens in reducing malaria transmission. In multivalent vaccines, and possibly even in OMV vaccines, when increasing the number of antigens, the possibility of antagonism between antibodies should be considered, as this can diminish the protective effects. For example, the reduction modifiable protein (Rmp) antibody has been shown to suppress the bactericidal activity of other antibodies (48). Therefore, it is necessary to optimize and screen the antigen combinations in subsequent research of multivalent vaccines to obtain the best protective effect and ensure no interference between antibodies. In addition, since the majority of *N. gonorrhoeae* strains exist *in vivo* as sialylated bacteria and the salivated *N. gonorrhoeae* develops resistance to the bactericidal activity of normal human serum (30, 49), which may be related to the addition of sialic acid residues that block the binding of anti-LOS antibodies present in normal human serum to bacteria. Salivated gonococci have also been found to be resistant to bactericidal effects of immune sera in addition to LOS (50). Our results show that sialylated gonococci are able to resist killing by several antisera to a certain extent, but sialylation did not affect the binding of antisera to bacteria. Wetzler et al. also found no difference in the binding of anti-PI monoclonal antibodies against surface exposed cyclic epitopes to sialylated and non-sialylated *N. gonorrhoeae* (50). Therefore, we speculate that the attenuation of antiserum bactericidal activity by sialylation may have affected the activation of the complement pathway. LOS sialylation is thought to potentially interfere with the ability of Ab to bind C1q (51). Sialic acids present on glycolipids or bacterial capsules can inhibit the complement cascade (52, 53), the bactericidal effect of anti-gonococcal antibodies is dependent on the complement cascade, and LOS sialylation may have an impact on their bactericidal effect. Moreover, sialylation of gonococcal LOS enhances factor H binding, thereby inhibiting alternative pathways (31, 51). LOS sialylation also inhibited the binding of serum mannose-binding lectin (MBL) to *N. gonorrhoeae*, thereby inhibiting the MBL pathway (51, 54). However, the complement can only be activated when *N. gonorrhoeae* is co-incubated with purified MBL-MASP prior to serum addition; MBL is not involved in complement activation of *N. gonorrhoeae* in the presence of complete serum containing C1-inhibitor and α_2_-macroglobulin (55). In this study, we used complete serum as a complement source, so the role of the MBL pathway may be limited.

Adhesion to host cells is a key link between bacterial colonization, subsequent invasion, and infection (56). The colonization ability of bacteria largely depends on their resistance mechanisms to host mechanical and immune clearance (57). Therefore, bacterial adherence inhibition should be a goal when developing vaccines (58). For example, anti-NanAT1-TufT1-PlyD4 antisera can inhibit the adhesion of *Streptococcus pneumoniae* to A549 cells, and *in vivo* experiments have shown that it reduces *S. pneumoniae* colonization in the lungs (38). We found that when approximately 5 × 10^4^ N*. gonorrhoeae* cells were added to a monolayer of ME-180 cells in the presence of antiserum, the trivalent vaccine antiserum inhibited gonococcal adhesion to ME-180 cells significantly better than the monovalent vaccine antiserum. The bacterial dose was close to the estimated infective dose of *N. gonorrhoeae* in humans. Our results showed that the vaccine effectiveness for clearing gonococcal infections in animal experiments was in agreement with the results of *in vitro* antibody adhesion inhibition assays compared to those of bactericidal assays. This suggests that vaccine-induced antibodies can reduce the initial level of gonococcal colonization in the host.

T helper (Th) cells play a key role in gonococcal infections (59). In the present study, antibody subtype analysis suggested that the trivalent vaccine significantly induced lgG1-type antibody responses, suggesting the indirect induction of a Th2-type immune response bias, which may be related to the CTB adjuvant used. The use of a CTB adjuvant induces a Th2-type response bias (60, 61). Splenocyte cytokine analysis revealed that splenocytes in the trivalent vaccine group secreted significantly more IFN-γ and IL-17A, suggesting the activation of Th1 and Th17 cellular immune responses. The clearance of *N. gonorrhoeae* is associated with Th1 and Th17 responses. Blocking IL-17 leads to prolonged gonorrhea, indicating that the Th17 response is involved in *N. gonorrhoeae* clearance (62). Gonococcal infection experiments in IL-12-knockout (Th1-deficient) mice have revealed that accelerated *N. gonorrhoeae* clearance is Th1-dependent (19). An effective gonorrhea vaccine should, therefore, induce a Th1 polarization response in the genital tract of mice, which may confer greater protection (29). However, the Th17 response is less protective against gonococci than the Th1 response, which does not produce protective immunity (63). Song et al. screened several adjuvant types to identify the optimal Th1-polarizing adjuvant, CpG1826, which triggered a strong Th1-polarizing antigen-specific immune response and provided excellent protection against gonococcal infection in a mouse model of vaginal infection (29). Therefore, it is necessary to screen and optimize adjuvants in subsequent studies to better activate the Th1 polarization response against gonorrhea.

In conclusion, the trivalent vaccine was highly immunogenic and broadly protective compared to monovalent vaccines, with the trivalent-vaccine-specific antiserum inhibiting *N. gonorrhoeae* adhesion to ME-180 cells and effectively clearing *N. gonorrhoeae* from the genital tract of mice. There are some limitations of our study. For example, although the monovalent and trivalent vaccines induced a strong antibody response, they did not show a surprising advantage in gonococcal infection clearance compared with controls. The possible reason is that Th1-biased IgG2a and IgG2b antibodies can activate complement more effectively than Th2-biased IgG1 antibodies, thereby enhancing the bactericidal activity of serum against *N. gonorrhoeae* (64). Hence, subsequent studies should also be carried out to evaluate the protective effect of multivalent vaccines using Th1-biased adjuvants.

## Data Availability

The original contributions presented in the study are included in the article/**Supplementary Material**. Further inquiries can be directed to the corresponding author/s.
