# Supplementary material for: Intranasal trivalent candidate vaccine induces strong mucosal and systemic immune responses against Neisseria gonorrhoeae
Source: Front Immunol. 2024 Nov 26;15:1473193. doi: 10.3389/fimmu.2024.1473193 (PMC11628552; doi:10.3389/fimmu.2024.1473193)
Supplement: Supplementary file 1 [file DataSheet1.docx]

Supplementary Material

Intranasal trivalent candidate vaccine induces strong mucosal and systemic immune responses against Neisseria gonorrhoeae

**Supplementary Figures and Tables**


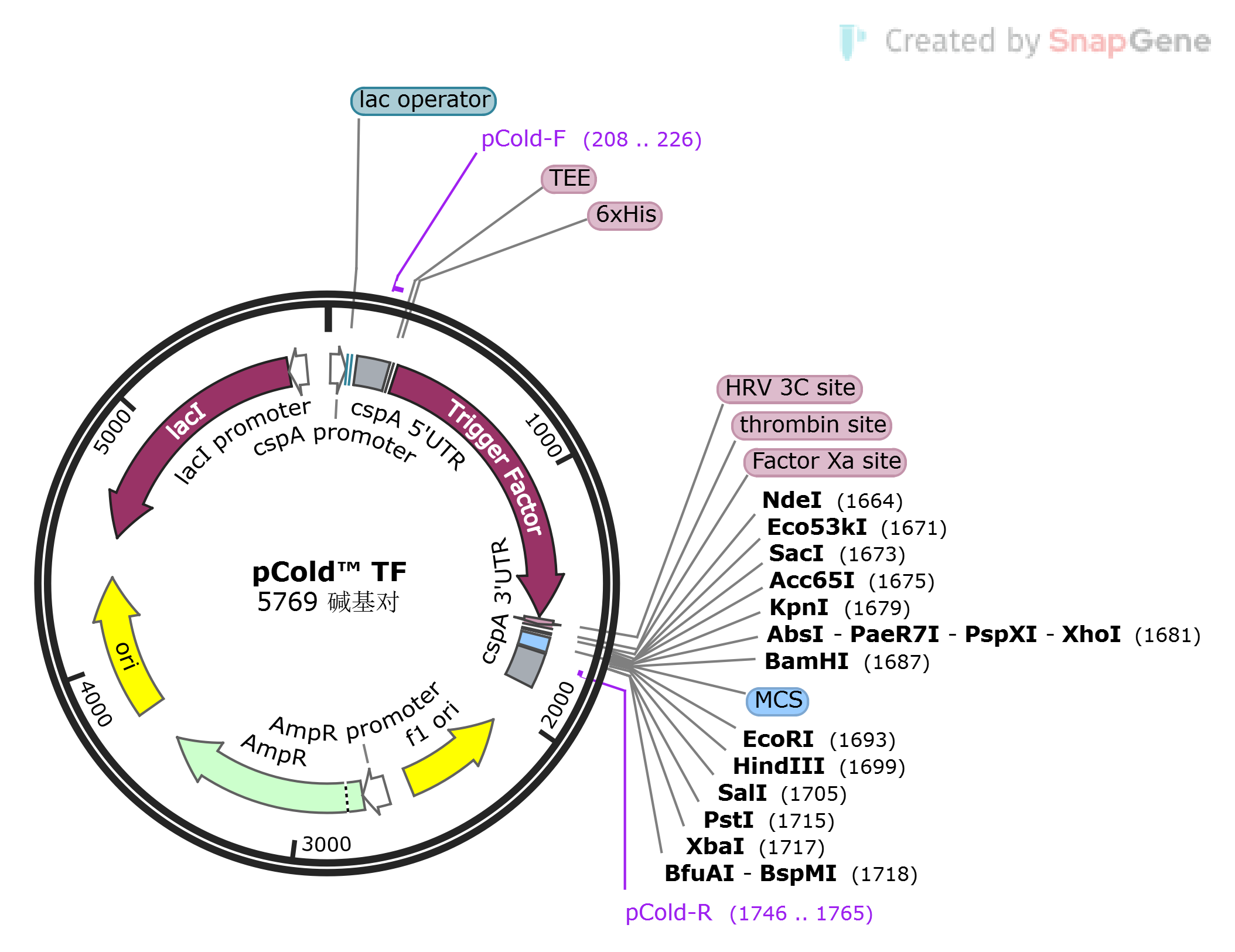


**Figure S1.** pCold TF plasmid map.

**
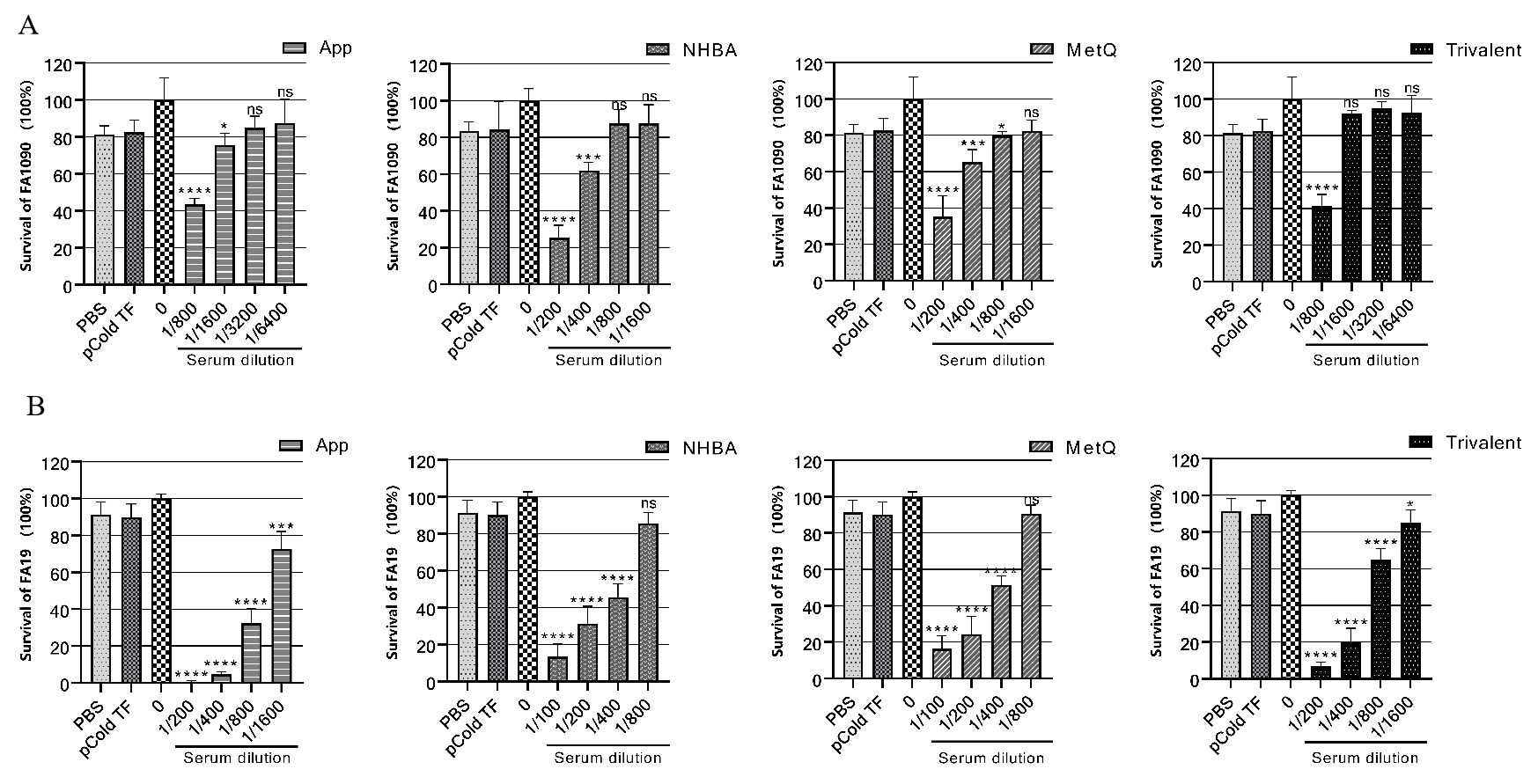
**

**Figure S2.** Analysis of antisera for bactericidal activity against *N. gonorrhoeae* strains FA1090 and FA19. **A-B**: Survival of strain FA1090 and FA19 in App, NHBA, MetQ, Trivalent, the untreated group (0, serum-free + complement-free) and lowest dilution control of PBS-immunized group antiserum (PBS, with complement) and pCold-TF-immunized group antiserum (pCold-TF, with complement) were set as controls. Data are representative of one of three independently repeated experiments. ns: non-significant, *P* > 0.05;**P ≤* 0.05; ***P ≤* 0.01; ****P ≤* 0.001; *****P ≤* 0.0001 (unpaired Student’s t-test).

**Table S1:** Sequences of gene primers (the underlined portion is the endonuclease position of *Hin*d III and *Xba* I respectively).

| Gene | Forword primer | Reverse primer |
| --- | --- | --- |
| Passenger | CCCAAGCTTGGACACACTTATTTCGGCATC | GCTCTAGAATTGGCATAACGGCTGA |
| NHBA | CCCAAGCTTATGTTTAAACGCAGTGTGATTGC | GCTCTAGATCAATCCCGATCTTTTTTGCCG |
| MetQ | CCCAAGCTTGGTCAAAAAGACAGCGCGCC | GCTCTAGATTATTTGGCTGCGCCTTCATTCC |

**Table S2** Conservativeness analysis of antigenic sequences (App was found in GenBank to be present in the following 100 genomes, with 99.93-100% nucleic acid identity).

| Description | Scientific Name | Max Score | Total Score | Query Cover | E value | Per. ident | Acc. Len | Accession |
| --- | --- | --- | --- | --- | --- | --- | --- | --- |
| Neisseria gonorrhoeae strain WHO_S_2024 chromosome, complete genome | Neisseria gonorrhoeae | 8139 | 8139 | 100% | 0 | 100 | 2221865 | CP145064.1 |
| Neisseria gonorrhoeae strain FA1090 N-1-60 chromosome, complete genome | Neisseria gonorrhoeae | 8139 | 8139 | 100% | 0 | 100 | 2155007 | CP115654.1 |
| Neisseria gonorrhoeae FA 1090, complete genome | Neisseria gonorrhoeae FA 1090 | 8139 | 8139 | 100% | 0 | 100 | 2153922 | AE004969.1 |
| Neisseria gonorrhoeae strain WHO_B_2024 chromosome, complete genome | Neisseria gonorrhoeae | 8133 | 8133 | 100% | 0 | 99.98 | 2219659 | CP145088.1 |
| Neisseria gonorrhoeae strain WHO_beta_2024 chromosome, complete genome | Neisseria gonorrhoeae | 8133 | 8133 | 100% | 0 | 99.98 | 2163258 | CP145053.1 |
| Neisseria gonorrhoeae strain WHO_alpha_2024 chromosome, complete genome | Neisseria gonorrhoeae | 8133 | 8133 | 100% | 0 | 99.98 | 2235802 | CP145057.1 |
| Neisseria gonorrhoeae strain 1123850 chromosome, complete genome | Neisseria gonorrhoeae | 8133 | 8133 | 100% | 0 | 99.98 | 2217711 | CP107271.1 |
| Neisseria gonorrhoeae strain ATCC 49226 chromosome, complete genome | Neisseria gonorrhoeae | 8133 | 8133 | 100% | 0 | 99.98 | 2162630 | CP098538.2 |
| Neisseria gonorrhoeae strain FQ82 chromosome, complete genome | Neisseria gonorrhoeae | 8128 | 8128 | 100% | 0 | 99.95 | 2217835 | CP034018.1 |
| Neisseria gonorrhoeae strain FQ48 chromosome, complete genome | Neisseria gonorrhoeae | 8128 | 8128 | 100% | 0 | 99.95 | 2229997 | CP034020.1 |
| Neisseria gonorrhoeae strain FQ04 chromosome, complete genome | Neisseria gonorrhoeae | 8128 | 8128 | 100% | 0 | 99.95 | 2217528 | CP034028.1 |
| Neisseria gonorrhoeae strain FQ84 chromosome, complete genome | Neisseria gonorrhoeae | 8128 | 8128 | 100% | 0 | 99.95 | 2217429 | CP034016.1 |
| Neisseria gonorrhoeae strain FDAARGOS_207 chromosome, complete genome | Neisseria gonorrhoeae | 8128 | 8128 | 100% | 0 | 99.95 | 2189300 | CP020419.2 |
| Neisseria gonorrhoeae NG-k51.05 chromosome, complete genome | Neisseria gonorrhoeae NG-k51.05 | 8128 | 8128 | 100% | 0 | 99.95 | 2232590 | CP003974.1 |
| Neisseria gonorrhoeae strain WHO_H_2024 chromosome, complete genome | Neisseria gonorrhoeae | 8128 | 8128 | 100% | 0 | 99.95 | 2233100 | CP145050.1 |
| Neisseria gonorrhoeae strain NCTC13800 genome assembly, chromosome: 1 | Neisseria gonorrhoeae | 8128 | 8128 | 100% | 0 | 99.95 | 2226638 | LT906472.1 |
| Neisseria gonorrhoeae strain NCTC13798 genome assembly, chromosome: 1 | Neisseria gonorrhoeae | 8128 | 8128 | 100% | 0 | 99.95 | 2230241 | LT906440.1 |
| Neisseria gonorrhoeae strain RIVM0640, complete genome | Neisseria gonorrhoeae | 8128 | 8128 | 100% | 0 | 99.95 | 2230041 | CP019467.1 |
| Neisseria gonorrhoeae strain 2004S05-027 chromosome, complete genome | Neisseria gonorrhoeae | 8128 | 8128 | 100% | 0 | 99.95 | 2226346 | CP131645.1 |
| Neisseria gonorrhoeae strain 2010C02-038 chromosome, complete genome | Neisseria gonorrhoeae | 8128 | 8128 | 100% | 0 | 99.95 | 2163486 | CP131641.1 |
| Neisseria gonorrhoeae strain 2016E02-233 chromosome, complete genome | Neisseria gonorrhoeae | 8128 | 8128 | 100% | 0 | 99.95 | 2221680 | CP131636.1 |
| Neisseria gonorrhoeae isolate 632_2023 genome assembly, chromosome: 1 | Neisseria gonorrhoeae | 8128 | 8128 | 100% | 0 | 99.95 | 2228442 | OZ004876.1 |
| Neisseria gonorrhoeae strain WHO_Y genome assembly, chromosome: 1 | Neisseria gonorrhoeae | 8128 | 8128 | 100% | 0 | 99.95 | 2228980 | LT592161.1 |
| Neisseria gonorrhoeae strain WHO_V genome assembly, chromosome: 1 | Neisseria gonorrhoeae | 8128 | 8128 | 100% | 0 | 99.95 | 2221284 | LT592150.1 |
| Neisseria gonorrhoeae strain WHO L genome assembly, chromosome: 1 | Neisseria gonorrhoeae | 8128 | 8128 | 100% | 0 | 99.95 | 2168633 | LT591901.1 |
| Neisseria gonorrhoeae strain 34530 chromosome, complete genome | Neisseria gonorrhoeae | 8128 | 8128 | 100% | 0 | 99.95 | 2228373 | CP016016.1 |
| Neisseria gonorrhoeae strain 2022NG-0032 plasmid p2022NG-0032_1, complete sequence | Neisseria gonorrhoeae | 8128 | 8128 | 100% | 0 | 99.95 | 2167441 | CP119142.1 |
| Neisseria gonorrhoeae strain FA6140, complete genome | Neisseria gonorrhoeae | 8128 | 8128 | 100% | 0 | 99.95 | 2168698 | CP012027.1 |
| Neisseria gonorrhoeae strain 9112 chromosome, complete genome | Neisseria gonorrhoeae | 8128 | 8128 | 100% | 0 | 99.95 | 2230440 | CP107266.1 |
| Neisseria gonorrhoeae strain 10272 chromosome, complete genome | Neisseria gonorrhoeae | 8128 | 8128 | 100% | 0 | 99.95 | 2219268 | CP106921.1 |
| Neisseria gonorrhoeae strain 10231 chromosome, complete genome | Neisseria gonorrhoeae | 8128 | 8128 | 100% | 0 | 99.95 | 2212810 | CP107264.1 |
| Neisseria gonorrhoeae strain 9071 chromosome, complete genome | Neisseria gonorrhoeae | 8128 | 8128 | 100% | 0 | 99.95 | 2263505 | CP106918.1 |
| Neisseria gonorrhoeae strain 1137292 chromosome | Neisseria gonorrhoeae | 8128 | 8128 | 100% | 0 | 99.95 | 2228866 | CP107273.1 |
| Neisseria gonorrhoeae strain 1130991 chromosome, complete genome | Neisseria gonorrhoeae | 8128 | 8128 | 100% | 0 | 99.95 | 2230471 | CP107272.1 |
| Neisseria gonorrhoeae strain 1081168 chromosome, complete genome | Neisseria gonorrhoeae | 8128 | 8128 | 100% | 0 | 99.95 | 2230282 | CP107270.1 |
| Neisseria gonorrhoeae strain 10239 chromosome, complete genome | Neisseria gonorrhoeae | 8128 | 8128 | 100% | 0 | 99.95 | 2223679 | CP098474.1 |
| Neisseria gonorrhoeae strain 10538 chromosome, complete genome | Neisseria gonorrhoeae | 8128 | 8128 | 100% | 0 | 99.95 | 2223795 | CP104548.2 |
| Neisseria gonorrhoeae strain 10269 chromosome, complete genome | Neisseria gonorrhoeae | 8128 | 8128 | 100% | 0 | 99.95 | 2214765 | CP098464.2 |
| Neisseria gonorrhoeae strain 10704 chromosome, complete genome | Neisseria gonorrhoeae | 8128 | 8128 | 100% | 0 | 99.95 | 2218708 | CP098542.2 |
| Neisseria gonorrhoeae strain 9464 chromosome, complete genome | Neisseria gonorrhoeae | 8128 | 8128 | 100% | 0 | 99.95 | 2228428 | CP106826.1 |
| Neisseria gonorrhoeae strain 9399 chromosome, complete genome | Neisseria gonorrhoeae | 8128 | 8128 | 100% | 0 | 99.95 | 2219165 | CP106824.1 |
| Neisseria gonorrhoeae strain 10529 chromosome, complete genome | Neisseria gonorrhoeae | 8128 | 8128 | 100% | 0 | 99.95 | 2211604 | CP106821.1 |
| Neisseria gonorrhoeae strain 10727 chromosome, complete genome | Neisseria gonorrhoeae | 8128 | 8128 | 100% | 0 | 99.95 | 2191505 | CP106777.1 |
| Neisseria gonorrhoeae strain 10500 chromosome, complete genome | Neisseria gonorrhoeae | 8128 | 8128 | 100% | 0 | 99.95 | 2214782 | CP106765.1 |
| Neisseria gonorrhoeae strain 10562 chromosome, complete genome | Neisseria gonorrhoeae | 8128 | 8128 | 100% | 0 | 99.95 | 2201001 | CP106773.1 |
| Neisseria gonorrhoeae strain 10702 chromosome, complete genome | Neisseria gonorrhoeae | 8128 | 8128 | 100% | 0 | 99.95 | 2212515 | CP106775.1 |
| Neisseria gonorrhoeae strain 10574 chromosome, complete genome | Neisseria gonorrhoeae | 8128 | 8128 | 100% | 0 | 99.95 | 2213959 | CP106817.1 |
| Neisseria gonorrhoeae strain 9431 chromosome, complete genome | Neisseria gonorrhoeae | 8128 | 8128 | 100% | 0 | 99.95 | 2227248 | CP106813.1 |
| Neisseria gonorrhoeae strain 10562 chromosome, complete genome | Neisseria gonorrhoeae | 8128 | 8128 | 100% | 0 | 99.95 | 2223176 | CP106815.1 |
| Neisseria gonorrhoeae strain 10819 chromosome, complete genome | Neisseria gonorrhoeae | 8128 | 8128 | 100% | 0 | 99.95 | 2229641 | CP106803.1 |
| Neisseria gonorrhoeae strain 10743 chromosome, complete genome | Neisseria gonorrhoeae | 8128 | 8128 | 100% | 0 | 99.95 | 2227249 | CP106805.1 |
| Neisseria gonorrhoeae strain 10744 chromosome, complete genome | Neisseria gonorrhoeae | 8128 | 8128 | 100% | 0 | 99.95 | 2228933 | CP106801.1 |
| Neisseria gonorrhoeae strain 10525 chromosome, complete genome | Neisseria gonorrhoeae | 8128 | 8128 | 100% | 0 | 99.95 | 2224751 | CP098534.2 |
| Neisseria gonorrhoeae strain 10537 chromosome, complete genome | Neisseria gonorrhoeae | 8128 | 8128 | 100% | 0 | 99.95 | 2228946 | CP098532.2 |
| Neisseria gonorrhoeae strain 10536 chromosome, complete genome | Neisseria gonorrhoeae | 8128 | 8128 | 100% | 0 | 99.95 | 2227438 | CP098505.2 |
| Neisseria gonorrhoeae strain 10268 chromosome, complete genome | Neisseria gonorrhoeae | 8128 | 8128 | 100% | 0 | 99.95 | 2210573 | CP098503.2 |
| Neisseria gonorrhoeae strain 9126 chromosome, complete genome | Neisseria gonorrhoeae | 8128 | 8128 | 100% | 0 | 99.95 | 2220893 | CP098546.2 |
| Neisseria gonorrhoeae strain 10720 chromosome, complete genome | Neisseria gonorrhoeae | 8128 | 8128 | 100% | 0 | 99.95 | 2219837 | CP098466.2 |
| Neisseria gonorrhoeae strain 9343 chromosome, complete genome | Neisseria gonorrhoeae | 8128 | 8128 | 100% | 0 | 99.95 | 2217931 | CP098548.2 |
| Neisseria gonorrhoeae strain 10577 chromosome, complete genome | Neisseria gonorrhoeae | 8128 | 8128 | 100% | 0 | 99.95 | 2214469 | CP098540.1 |
| Neisseria gonorrhoeae strain 9458 chromosome, complete genome | Neisseria gonorrhoeae | 8128 | 8128 | 100% | 0 | 99.95 | 2233050 | CP098476.1 |
| Neisseria gonorrhoeae strain 9460 chromosome, complete genome | Neisseria gonorrhoeae | 8128 | 8128 | 100% | 0 | 99.95 | 2228516 | CP098462.1 |
| Neisseria gonorrhoeae strain 98D159 chromosome, complete genome | Neisseria gonorrhoeae | 8128 | 8128 | 100% | 0 | 99.95 | 2172572 | CP078114.1 |
| Neisseria gonorrhoeae strain O2D156 chromosome, complete genome | Neisseria gonorrhoeae | 8128 | 8128 | 100% | 0 | 99.95 | 2165933 | CP078113.1 |
| Neisseria gonorrhoeae strain 3648 chromosome | Neisseria gonorrhoeae | 8128 | 8128 | 100% | 0 | 99.95 | 2214431 | CP040394.1 |
| Neisseria gonorrhoeae strain WHO_A_2024 chromosome, complete genome | Neisseria gonorrhoeae | 8122 | 8122 | 100% | 0 | 99.93 | 2308468 | CP145090.1 |
| Neisseria gonorrhoeae strain WHO_I_2024 chromosome, complete genome | Neisseria gonorrhoeae | 8122 | 8122 | 100% | 0 | 99.93 | 2171276 | CP145074.1 |
| Neisseria gonorrhoeae strain WHO_J_2024 chromosome, complete genome | Neisseria gonorrhoeae | 8122 | 8122 | 100% | 0 | 99.93 | 2168993 | CP145071.1 |
| Neisseria gonorrhoeae strain WHO_R_2024 chromosome, complete genome | Neisseria gonorrhoeae | 8122 | 8122 | 100% | 0 | 99.93 | 2218559 | CP145028.1 |
| Neisseria gonorrhoeae strain 2018S05-288 chromosome, complete genome | Neisseria gonorrhoeae | 8122 | 8122 | 100% | 0 | 99.93 | 2178594 | CP131632.1 |
| Neisseria gonorrhoeae strain 2020S05-258 chromosome, complete genome | Neisseria gonorrhoeae | 8122 | 8122 | 100% | 0 | 99.93 | 2235875 | CP131626.1 |
| Neisseria gonorrhoeae strain 2020N07-265 chromosome, complete genome | Neisseria gonorrhoeae | 8122 | 8122 | 100% | 0 | 99.93 | 2184061 | CP131622.1 |
| Neisseria gonorrhoeae isolate SGC-23-001 genome assembly, chromosome: contig1 | Neisseria gonorrhoeae | 8122 | 8122 | 100% | 0 | 99.93 | 2214561 | OZ021676.1 |
| Neisseria gonorrhoeae strain NJ195417 chromosome, complete genome | Neisseria gonorrhoeae | 8122 | 8122 | 100% | 0 | 99.93 | 2214712 | CP130899.1 |
| Neisseria gonorrhoeae strain NJ196610 chromosome, complete genome | Neisseria gonorrhoeae | 8122 | 8122 | 100% | 0 | 99.93 | 2216684 | CP130898.1 |
| Neisseria gonorrhoeae strain NJ1914215 chromosome, complete genome | Neisseria gonorrhoeae | 8122 | 8122 | 100% | 0 | 99.93 | 2216433 | CP130894.1 |
| Neisseria gonorrhoeae strain NJ1911400 chromosome, complete genome | Neisseria gonorrhoeae | 8122 | 8122 | 100% | 0 | 99.93 | 2216166 | CP130896.1 |
| Neisseria gonorrhoeae strain NJ1913940 chromosome, complete genome | Neisseria gonorrhoeae | 8122 | 8122 | 100% | 0 | 99.93 | 2213791 | CP130895.1 |
| Neisseria gonorrhoeae strain NJ197542 chromosome, complete genome | Neisseria gonorrhoeae | 8122 | 8122 | 100% | 0 | 99.93 | 2216731 | CP130897.1 |
| Neisseria gonorrhoeae strain NJ1914646 chromosome, complete genome | Neisseria gonorrhoeae | 8122 | 8122 | 100% | 0 | 99.93 | 2215504 | CP130893.1 |
| Neisseria gonorrhoeae strain NJ208756 chromosome, complete genome | Neisseria gonorrhoeae | 8122 | 8122 | 100% | 0 | 99.93 | 2214541 | CP130890.1 |
| Neisseria gonorrhoeae strain NJ208430 chromosome, complete genome | Neisseria gonorrhoeae | 8122 | 8122 | 100% | 0 | 99.93 | 2212965 | CP130891.1 |
| Neisseria gonorrhoeae strain NJ209649 chromosome, complete genome | Neisseria gonorrhoeae | 8122 | 8122 | 100% | 0 | 99.93 | 2216289 | CP130889.1 |
| Neisseria gonorrhoeae strain 1145734 chromosome, complete genome | Neisseria gonorrhoeae | 8122 | 8122 | 100% | 0 | 99.93 | 2163610 | CP107274.1 |
| Neisseria gonorrhoeae strain 10588 chromosome, complete genome | Neisseria gonorrhoeae | 8122 | 8122 | 100% | 0 | 99.93 | 2221044 | CP098458.1 |
| Neisseria gonorrhoeae strain 10612 chromosome, complete genome | Neisseria gonorrhoeae | 8122 | 8122 | 100% | 0 | 99.93 | 2220997 | CP098472.2 |
| Neisseria gonorrhoeae strain H035 chromosome, complete genome | Neisseria gonorrhoeae | 8122 | 8122 | 100% | 0 | 99.93 | 2167942 | CP107223.1 |
| Neisseria gonorrhoeae strain 10791 chromosome, complete genome | Neisseria gonorrhoeae | 8122 | 8122 | 100% | 0 | 99.93 | 2212689 | CP106819.1 |
| Neisseria gonorrhoeae strain 10638 chromosome, complete genome | Neisseria gonorrhoeae | 8122 | 8122 | 100% | 0 | 99.93 | 2204219 | CP106789.1 |
| Neisseria gonorrhoeae strain 10610 chromosome, complete genome | Neisseria gonorrhoeae | 8122 | 8122 | 100% | 0 | 99.93 | 2221786 | CP106767.1 |
| Neisseria gonorrhoeae strain 10524 chromosome, complete genome | Neisseria gonorrhoeae | 8122 | 8122 | 100% | 0 | 99.93 | 2207788 | CP106769.1 |
| Neisseria gonorrhoeae strain 10794 chromosome, complete genome | Neisseria gonorrhoeae | 8122 | 8122 | 100% | 0 | 99.93 | 2212713 | CP106780.1 |
| Neisseria gonorrhoeae strain 10531 chromosome, complete genome | Neisseria gonorrhoeae | 8122 | 8122 | 100% | 0 | 99.93 | 2214953 | CP106771.1 |
| Neisseria gonorrhoeae strain 10795 chromosome, complete genome | Neisseria gonorrhoeae | 8122 | 8122 | 100% | 0 | 99.93 | 2213168 | CP106782.1 |
| Neisseria gonorrhoeae strain 10531 chromosome, complete genome | Neisseria gonorrhoeae | 8122 | 8122 | 100% | 0 | 99.93 | 2214951 | CP106811.1 |
| Neisseria gonorrhoeae strain 10708 chromosome, complete genome | Neisseria gonorrhoeae | 8122 | 8122 | 100% | 0 | 99.93 | 2227633 | CP106809.1 |
| Neisseria gonorrhoeae strain 10723 chromosome, complete genome | Neisseria gonorrhoeae | 8122 | 8122 | 100% | 0 | 99.93 | 2192371 | CP106749.1 |
| Neisseria gonorrhoeae strain 10328 chromosome, complete genome | Neisseria gonorrhoeae | 8122 | 8122 | 100% | 0 | 99.93 | 2213728 | CP098468.2 |

**Table S3** Conservativeness analysis of antigenic sequences (NHBA was found in GenBank to be present in the following 100 genomes, with 96.37-100% nucleic acid identity).

| Description | Scientific Name | Max Score | Total Score | Query Cover | E value | Per. ident | Acc. Len | Accession |
| --- | --- | --- | --- | --- | --- | --- | --- | --- |
| Neisseria gonorrhoeae strain WHO_C_2024 chromosome, complete genome | Neisseria gonorrhoeae | 2383 | 2383 | 100% | 0 | 100 | 2167377 | CP145086.1 |
| Neisseria gonorrhoeae strain FA6140, complete genome | Neisseria gonorrhoeae | 2383 | 2383 | 100% | 0 | 100 | 2168698 | CP012027.1 |
| Neisseria gonorrhoeae strain FA1090 N-1-60 chromosome, complete genome | Neisseria gonorrhoeae | 2383 | 2383 | 100% | 0 | 100 | 2155007 | CP115654.1 |
| Neisseria gonorrhoeae FA 1090, complete genome | Neisseria gonorrhoeae FA 1090 | 2383 | 2383 | 100% | 0 | 100 | 2153922 | AE004969.1 |
| Neisseria gonorrhoeae strain NG-F62 GNA2132 (gna2132) gene, complete cds | Neisseria gonorrhoeae | 2383 | 2383 | 100% | 0 | 100 | 1290 | AF235146.1 |
| Neisseria gonorrhoeae strain WHO_beta_2024 chromosome, complete genome | Neisseria gonorrhoeae | 2377 | 2377 | 100% | 0 | 99.92 | 2163258 | CP145053.1 |
| Neisseria gonorrhoeae strain WHO L genome assembly, chromosome: 1 | Neisseria gonorrhoeae | 2377 | 2377 | 100% | 0 | 99.92 | 2168633 | LT591901.1 |
| Neisseria gonorrhoeae strain SW0032 chromosome | Neisseria gonorrhoeae | 2366 | 2366 | 100% | 0 | 99.77 | 2151426 | CP061487.1 |
| Neisseria gonorrhoeae strain SW0015 chromosome | Neisseria gonorrhoeae | 2361 | 2361 | 100% | 0 | 99.69 | 2153514 | CP061486.1 |
| Neisseria gonorrhoeae strain SW0011 chromosome | Neisseria gonorrhoeae | 2350 | 2350 | 100% | 0 | 99.53 | 2153173 | CP061488.1 |
| Neisseria gonorrhoeae strain SW0005 chromosome | Neisseria gonorrhoeae | 2344 | 2344 | 100% | 0 | 99.46 | 2152607 | CP061490.1 |
| Neisseria gonorrhoeae strain ATCC 49226 chromosome, complete genome | Neisseria gonorrhoeae | 2337 | 2337 | 100% | 0 | 99.46 | 2162630 | CP098538.2 |
| Neisseria gonorrhoeae strain ATCC 49226 chromosome | Neisseria gonorrhoeae | 2337 | 2337 | 100% | 0 | 99.46 | 2069529 | CP045728.1 |
| Neisseria gonorrhoeae strain SW0018 chromosome | Neisseria gonorrhoeae | 2322 | 2322 | 100% | 0 | 99.15 | 2147640 | CP061489.1 |
| Neisseria gonorrhoeae strain SW0002 chromosome | Neisseria gonorrhoeae | 2217 | 2217 | 100% | 0 | 97.67 | 2153658 | CP061491.1 |
| Neisseria gonorrhoeae strain SW0259 chromosome | Neisseria gonorrhoeae | 2189 | 2189 | 100% | 0 | 97.3 | 2150148 | CP061484.1 |
| Neisseria gonorrhoeae strain SW0223 chromosome | Neisseria gonorrhoeae | 2178 | 2178 | 100% | 0 | 97.14 | 2152289 | CP061492.1 |
| Neisseria gonorrhoeae strain SW0236 chromosome | Neisseria gonorrhoeae | 2161 | 2161 | 100% | 0 | 96.9 | 2148016 | CP061485.1 |
| Neisseria gonorrhoeae strain SW0274 chromosome | Neisseria gonorrhoeae | 2145 | 2145 | 100% | 0 | 96.67 | 2147987 | CP061483.1 |
| Neisseria gonorrhoeae strain SE690. chromosome, complete genome | Neisseria gonorrhoeae | 2135 | 2135 | 100% | 0 | 96.59 | 2170090 | CP116342.1 |
| Neisseria gonorrhoeae strain CT532 chromosome, complete genome | Neisseria gonorrhoeae | 2135 | 2135 | 100% | 0 | 96.59 | 2165075 | CP048250.1 |
| Neisseria gonorrhoeae strain WHO N genome assembly, chromosome: 1 | Neisseria gonorrhoeae | 2124 | 2124 | 100% | 0 | 96.44 | 2172826 | LT591910.1 |
| Neisseria gonorrhoeae strain WHO G genome assembly, chromosome: 1 | Neisseria gonorrhoeae | 2124 | 2124 | 100% | 0 | 96.44 | 2167361 | LT591898.1 |
| Neisseria gonorrhoeae strain MS11 HL-1-22 chromosome, complete genome | Neisseria gonorrhoeae | 2124 | 2124 | 100% | 0 | 96.44 | 2259189 | CP115904.1 |
| Neisseria gonorrhoeae strain MS11 chromosome, complete genome | Neisseria gonorrhoeae | 2124 | 2124 | 100% | 0 | 96.44 | 2234079 | CP078118.1 |
| Neisseria gonorrhoeae MS11, complete genome | Neisseria gonorrhoeae MS11 | 2124 | 2124 | 100% | 0 | 96.44 | 2233640 | CP003909.1 |
| Neisseria gonorrhoeae strain AUSMDU00010541 chromosome, complete genome | Neisseria gonorrhoeae | 2119 | 2119 | 100% | 0 | 96.45 | 2174817 | CP045832.1 |
| Neisseria gonorrhoeae isolate G7944 genome assembly, chromosome: I | Neisseria gonorrhoeae | 2119 | 2119 | 100% | 0 | 96.45 | 2172803 | LS999566.1 |
| Neisseria gonorrhoeae isolate G97687 genome assembly, chromosome: I | Neisseria gonorrhoeae | 2119 | 2119 | 100% | 0 | 96.45 | 2174841 | LS999565.1 |
| Neisseria gonorrhoeae strain FDAARGOS_207 chromosome, complete genome | Neisseria gonorrhoeae | 2119 | 2119 | 100% | 0 | 96.44 | 2189300 | CP020419.2 |
| Neisseria gonorrhoeae strain FDAARGOS_204 chromosome, complete genome | Neisseria gonorrhoeae | 2119 | 2119 | 100% | 0 | 96.44 | 2212454 | CP020415.2 |
| Neisseria gonorrhoeae strain WHO_Q_2024 chromosome, complete genome | Neisseria gonorrhoeae | 2119 | 2119 | 100% | 0 | 96.45 | 2177981 | CP145032.1 |
| Neisseria gonorrhoeae strain 2010C02-038 chromosome, complete genome | Neisseria gonorrhoeae | 2119 | 2119 | 100% | 0 | 96.44 | 2163486 | CP131641.1 |
| Neisseria gonorrhoeae strain WHO O genome assembly, chromosome: 1 | Neisseria gonorrhoeae | 2119 | 2119 | 100% | 0 | 96.44 | 2169062 | LT592146.1 |
| Neisseria gonorrhoeae strain NJ204705 chromosome, complete genome | Neisseria gonorrhoeae | 2119 | 2119 | 100% | 0 | 96.45 | 2239705 | CP130892.1 |
| Neisseria gonorrhoeae strain 10272 chromosome, complete genome | Neisseria gonorrhoeae | 2119 | 2119 | 100% | 0 | 96.44 | 2219268 | CP106921.1 |
| Neisseria gonorrhoeae strain 10231 chromosome, complete genome | Neisseria gonorrhoeae | 2119 | 2119 | 100% | 0 | 96.44 | 2212810 | CP107264.1 |
| Neisseria gonorrhoeae strain 9071 chromosome, complete genome | Neisseria gonorrhoeae | 2119 | 2119 | 100% | 0 | 96.44 | 2263505 | CP106918.1 |
| Neisseria gonorrhoeae strain 10588 chromosome, complete genome | Neisseria gonorrhoeae | 2119 | 2119 | 100% | 0 | 96.44 | 2221044 | CP098458.1 |
| Neisseria gonorrhoeae strain 10612 chromosome, complete genome | Neisseria gonorrhoeae | 2119 | 2119 | 100% | 0 | 96.44 | 2220997 | CP098472.2 |
| Neisseria gonorrhoeae strain 10269 chromosome, complete genome | Neisseria gonorrhoeae | 2119 | 2119 | 100% | 0 | 96.44 | 2214765 | CP098464.2 |
| Neisseria gonorrhoeae strain 10704 chromosome, complete genome | Neisseria gonorrhoeae | 2119 | 2119 | 100% | 0 | 96.44 | 2218708 | CP098542.2 |
| Neisseria gonorrhoeae strain 9399 chromosome, complete genome | Neisseria gonorrhoeae | 2119 | 2119 | 100% | 0 | 96.44 | 2219165 | CP106824.1 |
| Neisseria gonorrhoeae strain 10529 chromosome, complete genome | Neisseria gonorrhoeae | 2119 | 2119 | 100% | 0 | 96.44 | 2211604 | CP106821.1 |
| Neisseria gonorrhoeae strain 10791 chromosome, complete genome | Neisseria gonorrhoeae | 2119 | 2119 | 100% | 0 | 96.44 | 2212689 | CP106819.1 |
| Neisseria gonorrhoeae strain 10727 chromosome, complete genome | Neisseria gonorrhoeae | 2119 | 2119 | 100% | 0 | 96.44 | 2191505 | CP106777.1 |
| Neisseria gonorrhoeae strain 10638 chromosome, complete genome | Neisseria gonorrhoeae | 2119 | 2119 | 100% | 0 | 96.44 | 2204219 | CP106789.1 |
| Neisseria gonorrhoeae strain 10610 chromosome, complete genome | Neisseria gonorrhoeae | 2119 | 2119 | 100% | 0 | 96.44 | 2221786 | CP106767.1 |
| Neisseria gonorrhoeae strain 10500 chromosome, complete genome | Neisseria gonorrhoeae | 2119 | 2119 | 100% | 0 | 96.44 | 2214782 | CP106765.1 |
| Neisseria gonorrhoeae strain 10524 chromosome, complete genome | Neisseria gonorrhoeae | 2119 | 2119 | 100% | 0 | 96.44 | 2207788 | CP106769.1 |
| Neisseria gonorrhoeae strain 10562 chromosome, complete genome | Neisseria gonorrhoeae | 2119 | 2119 | 100% | 0 | 96.44 | 2201001 | CP106773.1 |
| Neisseria gonorrhoeae strain 10702 chromosome, complete genome | Neisseria gonorrhoeae | 2119 | 2119 | 100% | 0 | 96.44 | 2212515 | CP106775.1 |
| Neisseria gonorrhoeae strain 10794 chromosome, complete genome | Neisseria gonorrhoeae | 2119 | 2119 | 100% | 0 | 96.44 | 2212713 | CP106780.1 |
| Neisseria gonorrhoeae strain 10531 chromosome, complete genome | Neisseria gonorrhoeae | 2119 | 2119 | 100% | 0 | 96.44 | 2214953 | CP106771.1 |
| Neisseria gonorrhoeae strain 10795 chromosome, complete genome | Neisseria gonorrhoeae | 2119 | 2119 | 100% | 0 | 96.44 | 2213168 | CP106782.1 |
| Neisseria gonorrhoeae strain 10574 chromosome, complete genome | Neisseria gonorrhoeae | 2119 | 2119 | 100% | 0 | 96.44 | 2213959 | CP106817.1 |
| Neisseria gonorrhoeae strain 10562 chromosome, complete genome | Neisseria gonorrhoeae | 2119 | 2119 | 100% | 0 | 96.44 | 2223176 | CP106815.1 |
| Neisseria gonorrhoeae strain 10531 chromosome, complete genome | Neisseria gonorrhoeae | 2119 | 2119 | 100% | 0 | 96.44 | 2214951 | CP106811.1 |
| Neisseria gonorrhoeae strain 10723 chromosome, complete genome | Neisseria gonorrhoeae | 2119 | 2119 | 100% | 0 | 96.44 | 2192371 | CP106749.1 |
| Neisseria gonorrhoeae strain 10268 chromosome, complete genome | Neisseria gonorrhoeae | 2119 | 2119 | 100% | 0 | 96.44 | 2210573 | CP098503.2 |
| Neisseria gonorrhoeae strain 10792 chromosome, complete genome | Neisseria gonorrhoeae | 2119 | 2119 | 100% | 0 | 96.44 | 2214415 | CP098470.2 |
| Neisseria gonorrhoeae strain 10328 chromosome, complete genome | Neisseria gonorrhoeae | 2119 | 2119 | 100% | 0 | 96.44 | 2213728 | CP098468.2 |
| Neisseria gonorrhoeae strain 9126 chromosome, complete genome | Neisseria gonorrhoeae | 2119 | 2119 | 100% | 0 | 96.44 | 2220893 | CP098546.2 |
| Neisseria gonorrhoeae strain 10720 chromosome, complete genome | Neisseria gonorrhoeae | 2119 | 2119 | 100% | 0 | 96.44 | 2219837 | CP098466.2 |
| Neisseria gonorrhoeae strain 9343 chromosome, complete genome | Neisseria gonorrhoeae | 2119 | 2119 | 100% | 0 | 96.44 | 2217931 | CP098548.2 |
| Neisseria gonorrhoeae strain 10577 chromosome, complete genome | Neisseria gonorrhoeae | 2119 | 2119 | 100% | 0 | 96.44 | 2214469 | CP098540.1 |
| Neisseria gonorrhoeae strain 10771 chromosome, complete genome | Neisseria gonorrhoeae | 2119 | 2119 | 100% | 0 | 96.44 | 2210202 | CP098536.1 |
| Neisseria gonorrhoeae strain 10814 chromosome, complete genome | Neisseria gonorrhoeae | 2119 | 2119 | 100% | 0 | 96.44 | 2212315 | CP098460.1 |
| Neisseria gonorrhoeae strain 2312 chromosome | Neisseria gonorrhoeae | 2119 | 2119 | 100% | 0 | 96.44 | 2210720 | CP097461.1 |
| Neisseria gonorrhoeae strain 4020 chromosome | Neisseria gonorrhoeae | 2119 | 2119 | 100% | 0 | 96.44 | 2203166 | CP040395.1 |
| Neisseria gonorrhoeae strain WHO_alpha_2024 chromosome, complete genome | Neisseria gonorrhoeae | 2115 | 2115 | 100% | 0 | 96.37 | 2235802 | CP145057.1 |
| Neisseria gonorrhoeae strain WHO_I_2024 chromosome, complete genome | Neisseria gonorrhoeae | 2113 | 2113 | 100% | 0 | 96.37 | 2171276 | CP145074.1 |
| Neisseria gonorrhoeae strain WHO_T_2024 chromosome, complete genome | Neisseria gonorrhoeae | 2113 | 2113 | 100% | 0 | 96.37 | 2247111 | CP145062.1 |
| Neisseria gonorrhoeae strain WHO_S_2024 chromosome, complete genome | Neisseria gonorrhoeae | 2113 | 2113 | 100% | 0 | 96.37 | 2221865 | CP145064.1 |
| Neisseria gonorrhoeae strain WHO_S2_2024 chromosome, complete genome | Neisseria gonorrhoeae | 2113 | 2113 | 100% | 0 | 96.37 | 2172077 | CP145026.1 |
| Neisseria gonorrhoeae strain 2004S05-027 chromosome, complete genome | Neisseria gonorrhoeae | 2113 | 2113 | 100% | 0 | 96.37 | 2226346 | CP131645.1 |
| Neisseria gonorrhoeae strain 2018C07-301 chromosome, complete genome | Neisseria gonorrhoeae | 2113 | 2113 | 100% | 0 | 96.37 | 2234433 | CP131630.1 |
| Neisseria gonorrhoeae strain 2020N08-318 chromosome, complete genome | Neisseria gonorrhoeae | 2113 | 2113 | 100% | 0 | 96.37 | 2223600 | CP131620.1 |
| Neisseria gonorrhoeae isolate SGC-23-002 genome assembly, chromosome: contig1 | Neisseria gonorrhoeae | 2113 | 2113 | 100% | 0 | 96.37 | 2231477 | OZ021673.1 |
| Neisseria gonorrhoeae isolate 632_2023 genome assembly, chromosome: 1 | Neisseria gonorrhoeae | 2113 | 2113 | 100% | 0 | 96.37 | 2228442 | OZ004876.1 |
| Neisseria gonorrhoeae strain TH1713 chromosome, complete genome | Neisseria gonorrhoeae | 2113 | 2113 | 100% | 0 | 96.37 | 2228256 | CP138342.1 |
| Neisseria gonorrhoeae strain TH2288 chromosome, complete genome | Neisseria gonorrhoeae | 2113 | 2113 | 100% | 0 | 96.37 | 2227834 | CP138339.1 |
| Neisseria gonorrhoeae strain 2022NG-0076 plasmid p2022NG-0076_1, complete sequence | Neisseria gonorrhoeae | 2113 | 2113 | 100% | 0 | 96.37 | 2168363 | CP119140.1 |
| Neisseria gonorrhoeae strain 1123850 chromosome, complete genome | Neisseria gonorrhoeae | 2113 | 2113 | 100% | 0 | 96.37 | 2217711 | CP107271.1 |
| Neisseria gonorrhoeae strain 10239 chromosome, complete genome | Neisseria gonorrhoeae | 2113 | 2113 | 100% | 0 | 96.37 | 2223679 | CP098474.1 |
| Neisseria gonorrhoeae strain 9464 chromosome, complete genome | Neisseria gonorrhoeae | 2113 | 2113 | 100% | 0 | 96.37 | 2228428 | CP106826.1 |
| Neisseria gonorrhoeae strain 9431 chromosome, complete genome | Neisseria gonorrhoeae | 2113 | 2113 | 100% | 0 | 96.37 | 2227248 | CP106813.1 |
| Neisseria gonorrhoeae strain 10736 chromosome, complete genome | Neisseria gonorrhoeae | 2113 | 2113 | 100% | 0 | 96.37 | 2227374 | CP106807.1 |
| Neisseria gonorrhoeae strain 10708 chromosome, complete genome | Neisseria gonorrhoeae | 2113 | 2113 | 100% | 0 | 96.37 | 2227633 | CP106809.1 |
| Neisseria gonorrhoeae strain 10819 chromosome, complete genome | Neisseria gonorrhoeae | 2113 | 2113 | 100% | 0 | 96.37 | 2229641 | CP106803.1 |
| Neisseria gonorrhoeae strain 10743 chromosome, complete genome | Neisseria gonorrhoeae | 2113 | 2113 | 100% | 0 | 96.37 | 2227249 | CP106805.1 |
| Neisseria gonorrhoeae strain 10744 chromosome, complete genome | Neisseria gonorrhoeae | 2113 | 2113 | 100% | 0 | 96.37 | 2228933 | CP106801.1 |
| Neisseria gonorrhoeae strain 9458 chromosome, complete genome | Neisseria gonorrhoeae | 2113 | 2113 | 100% | 0 | 96.37 | 2233050 | CP098476.1 |
| Neisseria gonorrhoeae strain 9460 chromosome, complete genome | Neisseria gonorrhoeae | 2113 | 2113 | 100% | 0 | 96.37 | 2228516 | CP098462.1 |
| Neisseria gonorrhoeae strain AT159 chromosome, complete genome | Neisseria gonorrhoeae | 2113 | 2113 | 100% | 0 | 96.37 | 2232771 | CP097846.1 |
| Neisseria gonorrhoeae strain 351961 chromosome | Neisseria gonorrhoeae | 2113 | 2113 | 100% | 0 | 96.37 | 2242106 | CP097460.1 |
| Neisseria gonorrhoeae strain NG211 chromosome, complete genome | Neisseria gonorrhoeae | 2113 | 2113 | 100% | 0 | 96.37 | 2218825 | CP050930.1 |
| Neisseria gonorrhoeae strain 98D159 chromosome, complete genome | Neisseria gonorrhoeae | 2113 | 2113 | 100% | 0 | 96.37 | 2172572 | CP078114.1 |
| Neisseria gonorrhoeae strain SK92679 chromosome, complete genome | Neisseria gonorrhoeae | 2113 | 2113 | 100% | 0 | 96.37 | 2173187 | CP078117.1 |
| Neisseria gonorrhoeae strain O2D156 chromosome, complete genome | Neisseria gonorrhoeae | 2113 | 2113 | 100% | 0 | 96.37 | 2165933 | CP078113.1 |

**Table S4** Conservativeness analysis of antigenic sequences (MetQ was found in GenBank to be present in the following 100 genomes, with 99.88-100% nucleic acid identity).

| Description | Scientific Name | Max Score | Total Score | Query Cover | E value | Per. ident | Acc. Len | Accession |
| --- | --- | --- | --- | --- | --- | --- | --- | --- |
| Neisseria gonorrhoeae SS3160 DNA, complete genome | Neisseria gonorrhoeae | 1602 | 1602 | 100% | 0 | 100 | 2214955 | AP019853.2 |
| Neisseria gonorrhoeae strain NJ189125 chromosome, complete genome | Neisseria gonorrhoeae | 1602 | 1602 | 100% | 0 | 100 | 2185626 | CP041586.1 |
| Neisseria gonorrhoeae strain NJ1711654 chromosome, complete genome | Neisseria gonorrhoeae | 1602 | 1602 | 100% | 0 | 100 | 2215121 | CP041585.1 |
| Neisseria gonorrhoeae FC498 DNA, complete genome | Neisseria gonorrhoeae | 1602 | 1602 | 100% | 0 | 100 | 2216138 | AP018385.1 |
| Neisseria gonorrhoeae FC460 DNA, complete genome | Neisseria gonorrhoeae | 1602 | 1602 | 100% | 0 | 100 | 2215151 | AP018381.1 |
| Neisseria gonorrhoeae FC428 DNA, complete genome | Neisseria gonorrhoeae | 1602 | 1602 | 100% | 0 | 100 | 2218551 | AP018377.1 |
| Neisseria gonorrhoeae strain FQ48 chromosome, complete genome | Neisseria gonorrhoeae | 1602 | 1602 | 100% | 0 | 100 | 2229997 | CP034020.1 |
| Neisseria gonorrhoeae strain FDAARGOS_204 chromosome, complete genome | Neisseria gonorrhoeae | 1602 | 1602 | 100% | 0 | 100 | 2212454 | CP020415.2 |
| Neisseria gonorrhoeae strain WHO_B_2024 chromosome, complete genome | Neisseria gonorrhoeae | 1602 | 1602 | 100% | 0 | 100 | 2219659 | CP145088.1 |
| Neisseria gonorrhoeae strain WHO_E_2024 chromosome, complete genome | Neisseria gonorrhoeae | 1602 | 1602 | 100% | 0 | 100 | 2213585 | CP145080.1 |
| Neisseria gonorrhoeae strain WHO_S_2024 chromosome, complete genome | Neisseria gonorrhoeae | 1602 | 1602 | 100% | 0 | 100 | 2221865 | CP145064.1 |
| Neisseria gonorrhoeae strain WHO_H_2024 chromosome, complete genome | Neisseria gonorrhoeae | 1602 | 1602 | 100% | 0 | 100 | 2233100 | CP145050.1 |
| Neisseria gonorrhoeae strain WHO_R_2024 chromosome, complete genome | Neisseria gonorrhoeae | 1602 | 1602 | 100% | 0 | 100 | 2218559 | CP145028.1 |
| Neisseria gonorrhoeae strain NCTC13800 genome assembly, chromosome: 1 | Neisseria gonorrhoeae | 1602 | 1602 | 100% | 0 | 100 | 2226638 | LT906472.1 |
| Neisseria gonorrhoeae strain NCTC13798 genome assembly, chromosome: 1 | Neisseria gonorrhoeae | 1602 | 1602 | 100% | 0 | 100 | 2230241 | LT906440.1 |
| Neisseria gonorrhoeae strain RIVM0640, complete genome | Neisseria gonorrhoeae | 1602 | 1602 | 100% | 0 | 100 | 2230041 | CP019467.1 |
| Neisseria gonorrhoeae strain RIVM0610, complete genome | Neisseria gonorrhoeae | 1602 | 1602 | 100% | 0 | 100 | 2229240 | CP019466.1 |
| Neisseria gonorrhoeae isolate SGC-23-001 genome assembly, chromosome: contig1 | Neisseria gonorrhoeae | 1602 | 1602 | 100% | 0 | 100 | 2214561 | OZ021676.1 |
| Neisseria gonorrhoeae isolate 632_2023 genome assembly, chromosome: 1 | Neisseria gonorrhoeae | 1602 | 1602 | 100% | 0 | 100 | 2228442 | OZ004876.1 |
| Neisseria gonorrhoeae strain WHO_Y genome assembly, chromosome: 1 | Neisseria gonorrhoeae | 1602 | 1602 | 100% | 0 | 100 | 2228980 | LT592161.1 |
| Neisseria gonorrhoeae strain WHO_V genome assembly, chromosome: 1 | Neisseria gonorrhoeae | 1602 | 1602 | 100% | 0 | 100 | 2221284 | LT592150.1 |
| Neisseria gonorrhoeae strain NJ195417 chromosome, complete genome | Neisseria gonorrhoeae | 1602 | 1602 | 100% | 0 | 100 | 2214712 | CP130899.1 |
| Neisseria gonorrhoeae strain NJ196610 chromosome, complete genome | Neisseria gonorrhoeae | 1602 | 1602 | 100% | 0 | 100 | 2216684 | CP130898.1 |
| Neisseria gonorrhoeae strain NJ204705 chromosome, complete genome | Neisseria gonorrhoeae | 1602 | 1602 | 100% | 0 | 100 | 2239705 | CP130892.1 |
| Neisseria gonorrhoeae strain NJ1911400 chromosome, complete genome | Neisseria gonorrhoeae | 1602 | 1602 | 100% | 0 | 100 | 2216166 | CP130896.1 |
| Neisseria gonorrhoeae strain NJ1913940 chromosome, complete genome | Neisseria gonorrhoeae | 1602 | 1602 | 100% | 0 | 100 | 2213791 | CP130895.1 |
| Neisseria gonorrhoeae strain NJ197542 chromosome, complete genome | Neisseria gonorrhoeae | 1602 | 1602 | 100% | 0 | 100 | 2216731 | CP130897.1 |
| Neisseria gonorrhoeae strain NJ1914646 chromosome, complete genome | Neisseria gonorrhoeae | 1602 | 1602 | 100% | 0 | 100 | 2215504 | CP130893.1 |
| Neisseria gonorrhoeae strain NJ208756 chromosome, complete genome | Neisseria gonorrhoeae | 1602 | 1602 | 100% | 0 | 100 | 2214541 | CP130890.1 |
| Neisseria gonorrhoeae strain NJ208430 chromosome, complete genome | Neisseria gonorrhoeae | 1602 | 1602 | 100% | 0 | 100 | 2212965 | CP130891.1 |
| Neisseria gonorrhoeae strain 34769 chromosome, complete genome | Neisseria gonorrhoeae | 1602 | 1602 | 100% | 0 | 100 | 2220340 | CP016017.1 |
| Neisseria gonorrhoeae strain 34530 chromosome, complete genome | Neisseria gonorrhoeae | 1602 | 1602 | 100% | 0 | 100 | 2228373 | CP016016.1 |
| Neisseria gonorrhoeae strain 2022NG-0032 plasmid p2022NG-0032_1, complete sequence | Neisseria gonorrhoeae | 1602 | 1602 | 100% | 0 | 100 | 2167441 | CP119142.1 |
| Neisseria gonorrhoeae strain FA19, complete genome | Neisseria gonorrhoeae | 1602 | 1602 | 100% | 0 | 100 | 2232367 | CP012026.1 |
| Neisseria gonorrhoeae strain FA1090 N-1-60 chromosome, complete genome | Neisseria gonorrhoeae | 1602 | 1602 | 100% | 0 | 100 | 2155007 | CP115654.1 |
| Neisseria gonorrhoeae strain 10272 chromosome, complete genome | Neisseria gonorrhoeae | 1602 | 1602 | 100% | 0 | 100 | 2219268 | CP106921.1 |
| Neisseria gonorrhoeae strain 10231 chromosome, complete genome | Neisseria gonorrhoeae | 1602 | 1602 | 100% | 0 | 100 | 2212810 | CP107264.1 |
| Neisseria gonorrhoeae strain 9071 chromosome, complete genome | Neisseria gonorrhoeae | 1602 | 1602 | 100% | 0 | 100 | 2263505 | CP106918.1 |
| Neisseria gonorrhoeae strain 1137292 chromosome | Neisseria gonorrhoeae | 1602 | 1602 | 100% | 0 | 100 | 2228866 | CP107273.1 |
| Neisseria gonorrhoeae strain 1130991 chromosome, complete genome | Neisseria gonorrhoeae | 1602 | 1602 | 100% | 0 | 100 | 2230471 | CP107272.1 |
| Neisseria gonorrhoeae strain 1081168 chromosome, complete genome | Neisseria gonorrhoeae | 1602 | 1602 | 100% | 0 | 100 | 2230282 | CP107270.1 |
| Neisseria gonorrhoeae strain 10239 chromosome, complete genome | Neisseria gonorrhoeae | 1602 | 1602 | 100% | 0 | 100 | 2223679 | CP098474.1 |
| Neisseria gonorrhoeae strain 10588 chromosome, complete genome | Neisseria gonorrhoeae | 1602 | 1602 | 100% | 0 | 100 | 2221044 | CP098458.1 |
| Neisseria gonorrhoeae strain 10612 chromosome, complete genome | Neisseria gonorrhoeae | 1602 | 1602 | 100% | 0 | 100 | 2220997 | CP098472.2 |
| Neisseria gonorrhoeae strain 10269 chromosome, complete genome | Neisseria gonorrhoeae | 1602 | 1602 | 100% | 0 | 100 | 2214765 | CP098464.2 |
| Neisseria gonorrhoeae strain 10704 chromosome, complete genome | Neisseria gonorrhoeae | 1602 | 1602 | 100% | 0 | 100 | 2218708 | CP098542.2 |
| Neisseria gonorrhoeae strain 9464 chromosome, complete genome | Neisseria gonorrhoeae | 1602 | 1602 | 100% | 0 | 100 | 2228428 | CP106826.1 |
| Neisseria gonorrhoeae strain 9399 chromosome, complete genome | Neisseria gonorrhoeae | 1602 | 1602 | 100% | 0 | 100 | 2219165 | CP106824.1 |
| Neisseria gonorrhoeae strain 10791 chromosome, complete genome | Neisseria gonorrhoeae | 1602 | 1602 | 100% | 0 | 100 | 2212689 | CP106819.1 |
| Neisseria gonorrhoeae strain 10727 chromosome, complete genome | Neisseria gonorrhoeae | 1602 | 1602 | 100% | 0 | 100 | 2191505 | CP106777.1 |
| Neisseria gonorrhoeae strain 10638 chromosome, complete genome | Neisseria gonorrhoeae | 1602 | 1602 | 100% | 0 | 100 | 2204219 | CP106789.1 |
| Neisseria gonorrhoeae strain 10610 chromosome, complete genome | Neisseria gonorrhoeae | 1602 | 1602 | 100% | 0 | 100 | 2221786 | CP106767.1 |
| Neisseria gonorrhoeae strain 10500 chromosome, complete genome | Neisseria gonorrhoeae | 1602 | 1602 | 100% | 0 | 100 | 2214782 | CP106765.1 |
| Neisseria gonorrhoeae strain 10524 chromosome, complete genome | Neisseria gonorrhoeae | 1602 | 1602 | 100% | 0 | 100 | 2207788 | CP106769.1 |
| Neisseria gonorrhoeae strain 10562 chromosome, complete genome | Neisseria gonorrhoeae | 1602 | 1602 | 100% | 0 | 100 | 2201001 | CP106773.1 |
| Neisseria gonorrhoeae strain 10702 chromosome, complete genome | Neisseria gonorrhoeae | 1602 | 1602 | 100% | 0 | 100 | 2212515 | CP106775.1 |
| Neisseria gonorrhoeae strain 10794 chromosome, complete genome | Neisseria gonorrhoeae | 1602 | 1602 | 100% | 0 | 100 | 2212713 | CP106780.1 |
| Neisseria gonorrhoeae strain 10531 chromosome, complete genome | Neisseria gonorrhoeae | 1602 | 1602 | 100% | 0 | 100 | 2214953 | CP106771.1 |
| Neisseria gonorrhoeae strain 10795 chromosome, complete genome | Neisseria gonorrhoeae | 1602 | 1602 | 100% | 0 | 100 | 2213168 | CP106782.1 |
| Neisseria gonorrhoeae strain 10574 chromosome, complete genome | Neisseria gonorrhoeae | 1602 | 1602 | 100% | 0 | 100 | 2213959 | CP106817.1 |
| Neisseria gonorrhoeae strain 9431 chromosome, complete genome | Neisseria gonorrhoeae | 1602 | 1602 | 100% | 0 | 100 | 2227248 | CP106813.1 |
| Neisseria gonorrhoeae strain 10562 chromosome, complete genome | Neisseria gonorrhoeae | 1602 | 1602 | 100% | 0 | 100 | 2223176 | CP106815.1 |
| Neisseria gonorrhoeae strain 10531 chromosome, complete genome | Neisseria gonorrhoeae | 1602 | 1602 | 100% | 0 | 100 | 2214951 | CP106811.1 |
| Neisseria gonorrhoeae strain 10736 chromosome, complete genome | Neisseria gonorrhoeae | 1602 | 1602 | 100% | 0 | 100 | 2227374 | CP106807.1 |
| Neisseria gonorrhoeae strain 10708 chromosome, complete genome | Neisseria gonorrhoeae | 1602 | 1602 | 100% | 0 | 100 | 2227633 | CP106809.1 |
| Neisseria gonorrhoeae strain 10819 chromosome, complete genome | Neisseria gonorrhoeae | 1602 | 1602 | 100% | 0 | 100 | 2229641 | CP106803.1 |
| Neisseria gonorrhoeae strain 10743 chromosome, complete genome | Neisseria gonorrhoeae | 1602 | 1602 | 100% | 0 | 100 | 2227249 | CP106805.1 |
| Neisseria gonorrhoeae strain 10744 chromosome, complete genome | Neisseria gonorrhoeae | 1602 | 1602 | 100% | 0 | 100 | 2228933 | CP106801.1 |
| Neisseria gonorrhoeae strain 10723 chromosome, complete genome | Neisseria gonorrhoeae | 1602 | 1602 | 100% | 0 | 100 | 2192371 | CP106749.1 |
| Neisseria gonorrhoeae strain 10268 chromosome, complete genome | Neisseria gonorrhoeae | 1602 | 1602 | 100% | 0 | 100 | 2210573 | CP098503.2 |
| Neisseria gonorrhoeae strain 10792 chromosome, complete genome | Neisseria gonorrhoeae | 1602 | 1602 | 100% | 0 | 100 | 2214415 | CP098470.2 |
| Neisseria gonorrhoeae strain 10328 chromosome, complete genome | Neisseria gonorrhoeae | 1602 | 1602 | 100% | 0 | 100 | 2213728 | CP098468.2 |
| Neisseria gonorrhoeae strain 9126 chromosome, complete genome | Neisseria gonorrhoeae | 1602 | 1602 | 100% | 0 | 100 | 2220893 | CP098546.2 |
| Neisseria gonorrhoeae strain 10720 chromosome, complete genome | Neisseria gonorrhoeae | 1602 | 1602 | 100% | 0 | 100 | 2219837 | CP098466.2 |
| Neisseria gonorrhoeae strain 9343 chromosome, complete genome | Neisseria gonorrhoeae | 1602 | 1602 | 100% | 0 | 100 | 2217931 | CP098548.2 |
| Neisseria gonorrhoeae strain 10296 chromosome, complete genome | Neisseria gonorrhoeae | 1602 | 1602 | 100% | 0 | 100 | 2216240 | CP098544.1 |
| Neisseria gonorrhoeae strain 10577 chromosome, complete genome | Neisseria gonorrhoeae | 1602 | 1602 | 100% | 0 | 100 | 2214469 | CP098540.1 |
| Neisseria gonorrhoeae strain 10771 chromosome, complete genome | Neisseria gonorrhoeae | 1602 | 1602 | 100% | 0 | 100 | 2210202 | CP098536.1 |
| Neisseria gonorrhoeae strain 9458 chromosome, complete genome | Neisseria gonorrhoeae | 1602 | 1602 | 100% | 0 | 100 | 2233050 | CP098476.1 |
| Neisseria gonorrhoeae strain 9460 chromosome, complete genome | Neisseria gonorrhoeae | 1602 | 1602 | 100% | 0 | 100 | 2228516 | CP098462.1 |
| Neisseria gonorrhoeae strain 10814 chromosome, complete genome | Neisseria gonorrhoeae | 1602 | 1602 | 100% | 0 | 100 | 2212315 | CP098460.1 |
| Neisseria gonorrhoeae strain 652 chromosome | Neisseria gonorrhoeae | 1602 | 1602 | 100% | 0 | 100 | 2174453 | CP097459.1 |
| Neisseria gonorrhoeae strain 98D159 chromosome, complete genome | Neisseria gonorrhoeae | 1602 | 1602 | 100% | 0 | 100 | 2172572 | CP078114.1 |
| Neisseria gonorrhoeae strain SRRSH203 chromosome, complete genome | Neisseria gonorrhoeae | 1602 | 1602 | 100% | 0 | 100 | 2217387 | CP048908.1 |
| Neisseria gonorrhoeae strain SRRSH204 chromosome, complete genome | Neisseria gonorrhoeae | 1602 | 1602 | 100% | 0 | 100 | 2217364 | CP048911.1 |
| Neisseria gonorrhoeae strain SRRSH205 chromosome, complete genome | Neisseria gonorrhoeae | 1602 | 1602 | 100% | 0 | 100 | 2217377 | CP048905.1 |
| Neisseria gonorrhoeae strain SRRSH207 chromosome, complete genome | Neisseria gonorrhoeae | 1602 | 1602 | 100% | 0 | 100 | 2214666 | CP048902.1 |
| Neisseria gonorrhoeae strain SRRSH214 chromosome, complete genome | Neisseria gonorrhoeae | 1602 | 1602 | 100% | 0 | 100 | 2217337 | CP048899.1 |
| Neisseria gonorrhoeae strain SRRSH229 chromosome, complete genome | Neisseria gonorrhoeae | 1602 | 1602 | 100% | 0 | 100 | 2219936 | CP048896.1 |
| Neisseria gonorrhoeae strain SRRSH240 chromosome, complete genome | Neisseria gonorrhoeae | 1602 | 1602 | 100% | 0 | 100 | 2214660 | CP048893.1 |
| Neisseria gonorrhoeae strain SW0274 chromosome | Neisseria gonorrhoeae | 1602 | 1602 | 100% | 0 | 100 | 2147987 | CP061483.1 |
| Neisseria gonorrhoeae strain SW0259 chromosome | Neisseria gonorrhoeae | 1602 | 1602 | 100% | 0 | 100 | 2150148 | CP061484.1 |
| Neisseria gonorrhoeae strain SW0032 chromosome | Neisseria gonorrhoeae | 1602 | 1602 | 100% | 0 | 100 | 2151426 | CP061487.1 |
| Neisseria gonorrhoeae strain SW0005 chromosome | Neisseria gonorrhoeae | 1602 | 1602 | 100% | 0 | 100 | 2152607 | CP061490.1 |
| Neisseria gonorrhoeae strain BJ16148 chromosome, complete genome | Neisseria gonorrhoeae | 1602 | 1602 | 100% | 0 | 100 | 2215157 | CP050505.1 |
| Neisseria gonorrhoeae strain 3648 chromosome | Neisseria gonorrhoeae | 1602 | 1602 | 100% | 0 | 100 | 2214431 | CP040394.1 |
| Neisseria gonorrhoeae FA 1090, complete genome | Neisseria gonorrhoeae FA 1090 | 1602 | 1602 | 100% | 0 | 100 | 2153922 | AE004969.1 |
| Neisseria gonorrhoeae strain WHO_A_2024 chromosome, complete genome | Neisseria gonorrhoeae | 1596 | 1596 | 100% | 0 | 99.88 | 2308468 | CP145090.1 |
| Neisseria gonorrhoeae strain WHO_D_2024 chromosome, complete genome | Neisseria gonorrhoeae | 1596 | 1596 | 100% | 0 | 99.88 | 2178180 | CP145083.1 |
| Neisseria gonorrhoeae strain WHO_J_2024 chromosome, complete genome | Neisseria gonorrhoeae | 1596 | 1596 | 100% | 0 | 99.88 | 2168993 | CP145071.1 |
